# Competitive α-glucosidase inhibitors, dihydrobenzoxanthones, from the barks of *Artocarpus elasticus*

**DOI:** 10.1080/14756366.2019.1660653

**Published:** 2019-09-03

**Authors:** Janar Jenis, Aizhamal Baiseitova, Sang Hwa Yoon, Chanin Park, Jeong Yoon Kim, Zuo Peng Li, Keun Woo Lee, Ki Hun Park

**Affiliations:** a Research Center for Medicinal Plants, Al-Farabi Kazakh National University, Almaty, Kazakhstan;; b Division of Applied Life Science (BK21 plus), IALS, Gyeongsang National University, Jinju, Republic of Korea;; c Division of Applied Life Science (BK21 plus), PMBBRC, RINS, Gyeongsang National University, Jinju, Republic of Korea

**Keywords:** *Artocarpus elasticus*, dihydrobenzoxanthones, artoindonesianin W, artoflavone B and α-glucosidase inhibition

## Abstract

This study aimed to search the α-glucosidase inhibitors from the barks part of *Artocarpus elasticus*. The responsible compounds for α-glucosidase inhibition were found out as dihydrobenzoxanthones (**1**–**4**) and alkylated flavones (**5**–**6**). All compounds showed a significant enzyme inhibition toward α-glucosidase with IC_50_s of 7.6–25.4 μM. Dihydrobenzoxanthones (**1**–**4**) exhibited a competitive inhibition to α-glucosidase. This competitive behaviour was fully characterised by double reciprocal plots, Yang’s method, and time-dependent experiments. The compound **1** manifested as the competitive and reversible simple slow-binding, with kinetic parameters *k*_3_ = 0.0437 µM^−1 ^min^−1^, *k*_4_ = 0.0166 min^−1^, and $K_{i}^{\mathrm{app}}$= 0.3795 µM. Alkylated flavones (**5**–**6**) were mixed type I (*K*_I_ < *K*_IS_) inhibitors. The binding affinities (*K*_SV_) represented by all inhibitors were correlated to their concentrations and inhibitory potencies (IC_50_). Moreover, compounds **1** and **5** were identified as new ones named as artoindonesianin W and artoflavone B, respectively. Molecular modelling study proposed the putative binding conformation of competitive inhibitors (**1**–**4**) to α-glucosidase at the atomic level.

## Introduction

α-Glucosidase is a widespread enzyme responsible for the hydrolytic cleavage of glucosidic bonds, which involves a number of essentially biological processes from the digestive process of carbohydrate to glycoprotein assembly^1–3^. α-Glucosidase inhibitors might reduce a blood sugar level and lead to suppressed postprandial hyperglycaemia responsible for diabetes and obesity^4^^,^^5^. The surface of a mammalian cell is decorated with complex carbohydrates known as glycans^6^. Glycans mediate a cell’s communication with other cells and the outside world^7^. The pattern of complex cell-surface glycans gives each cell type a unique and reproducible identity^6^. These glycans are subjected to extensive modification as glycoprotein mature and move to their final destination by glycosidase. Most notable, α-glucosidase plays a more essential role because three terminals are units at fourteen sugars of Glc_2_Man_9_GluNAc_2_ on glycan^8^^,^^9^. Thus, the α-glucosidase inhibitory functions are associated with antitumor and antiviral substances^10^^,^^11^.

*Artocarpus elasticus*, known as Terap, belongs to the family of Moraceae and grows in the tropical regions of Asia. *Artocarpus* plants comprise about 50 species, and their fruits are popular in the market. Their roots and leaves parts have been used as a traditional medicine in Indonesia against inflammation, malarial fever, hypertension, and diabetes^12^. Most of the pharmacological effects can be explained by the phenolic compounds, including flavonoids, stilbenoid, and prenylated flavonoids. Individual metabolites in extracts showed antibacterial, antitubercular, antiviral, cytotoxic, antioxidative, tyrosinase and 5-α reductase inhibitory activities^13–16^. Especially, alkylated flavonoids from *A. elasticus* revealed significant cytotoxic effect against human cancer cell lines and antioxidant activities^17–19^.

The purpose of this study was to isolate α-glucosidase inhibitory compounds from the barks of *A. elasticus*, and the identification of their structures by using spectrometric data. Their inhibitory capacities and kinetics were fully characterised by double reciprocal plots, Yang’s method, and slow-binding experiments. Binding affinity levels between inhibitors and enzyme were also confirmed by using fluorescence. The specific binding sites of inhibitors on active site were elucidated by molecular docking experiment.

## Materials and methods

### Instruments and chemicals

^1^H and ^13 ^C NMR spectra were recorded on a Bruker AM500 spectrometer (Bruker, Karlsruhe, Germany). Melting points were measured on a Thomas Scientific Capillary Melting Point Apparatus. MS and HR-MS were obtained on a JEOL JMS-700 mass spectrometer (JEOL, Tokyo, Japan). IR spectra were recorded on Varian 640-IR (Varian, Inc., USA). Optical rotation was measured on a Perkin-Elmer 343 polarimeter (Perkin-Elmer, Bridgeport, USA). Recycled HPLC and MPLC were conducted on Forte/R 100 (YMC Co., Ltd., Kyoto, Japan) using Triart C18 (S-5 µm, 12 nm and S-10 µm, 12 nm, YMC, Japan). Analytical grade methanol, acetonitrile, and acetic acid for HPLC were purchased from Fisher (Fisher Scientific Korea Ltd.). UV spectra and enzymatic assays were carried out on a SpectraMax M3 Multi-Mode Microplate Reader (Molecular device, USA). α-Glucosidase (EC3.2.1.20) was purchased from Sigma Aldrich St. Louis, USA. All chemicals were of analytical grade.

### Plant material

The barks of *A. elasticus* were collected by associated professor Dr. Mohd Azlan Nafiah on December 2013 from Malaysia. A Voucher specimen (TM1016) was deposited in the Universiti Pendidikan Sultan Idris, Malaysia.

### Extraction and isolation

The dried barks (250 g) of *A. elasticus* were extracted using methanol (10 L) at room temperature to give the crude extract (27 g). The crude extract was suspended in water and successively fractionated into chloroform to afford a dark residue (14 g). The chloroform fraction was subjected to column chromatography on MCI GEL CHP20P (300 mm × 50.0 mm, 75–150 μm, 500 g) and eluted with gradient flow of water/methanol (8:2 to 0:1, v/v) to give 15 fractions (A1-15, each 1000 mL). Fractions A9-12 (4.6 g) were fractionated via MPLC (250 mm × 30.0 mm, S-10 μm, 12 nm, YMC) eluting with a gradual increase of MeOH (0–100%) in H_2_O to afford 80 subfractions (B1-80). The above MPLC process was repeated 0.5 g each time. The subfractions B26-35 (1.8 g) enriched with compounds (**1**, **2**, and **6**) were further chromatographed over recycle HPLC (250 mm × 30.0 mm, S-5 μm, 12 nm, YMC) to give compounds **1** (28 mg), **2** (19 mg), and **6** (24 mg). Similarly, the subfractions B36-43 (1.4 g) were carried out to recycle HPLC to afford compounds **3** (15 mg), **4** (21 mg), and **5** (18 mg).

### Artoindonesianin W (1)

Brown amorphous powder. Mp 168–170 °C. ${\left[\alpha \right]}_{D}^{20}$ +77.5 (c 0.1, MeOH). UV (MeOH) *λ*_max_ (log *ɛ*) 205 (4.02), 230 (4.08), 250 (4.08), 260 (4.07), 275 (4.05), 375 (4.02). IR (KBr) 3430, 2927, 1655, 1598 cm^−1^. FABMS, *m*/*z* 383 [M + H]^+^; HRFABMS, *m*/*z* 383.1149 [M + H]^+^ (calcd for C_21_H_19_O_7_ 383.1056). ^1^H NMR (300 MHz, acetone-*d_6_*) *δ* 1.78 (3H, s, H-12), 2.43 (1H, dd, *J* = 15.98 Hz, H-9a), 3.39 (1H, dd, *J* = 15.98 Hz, H-9b), 3.89 (3H, s, 7-OCH_3_), 3.99 (1H, d, *J* = 6.3 Hz, H-10), 4.29 (1H, s, H-13a), 4.65 (1H, s, H-13b), 6.29 (1H, s, H-6), 6.50 (1H, s, H-3'), 6.65 (1H, s, H-8). ^13 ^C NMR (125 MHz, acetone-*d_6_*) δ 21.97 (C-12), 22.39 (C-9), 38.01 (C-10), 56.39 (7-OCH_3_), 93.17 (C-8), 98.68 (C-6), 103.84 (C-3'), 105.64 (C-4a), 106.56 (C-1'), 111.67 (C-3), 111.88 (C-13), 129.42 (C-6'), 136.93 (C-5'), 145.37 (C-11), 151.16 (C-4'), 151.40 (C-2'), 157.67 (C-8a), 161.85 (C-2), 162.92 (C-5), 166.09 (C-7), 181.09 (C = O, C-4) (see Figures S1, S2, and S7 in Supplementary Material).

### Artobiloxanthone (2)

Brown gum; EIMS, *m*/*z* 434 [M]^+^; HREIMS, *m*/*z* 434.1363 [M + H]^+^ (calcd for C_25_H_22_O_7_ 434.1366); ^1^H NMR (500 MHz, acetone-*d_6_*) *δ* 1.51 (3H, s, H-17), 1.54 (3H, s, H-18), 1.86 (3H, s, H-13), 2.51 (1H, dd, *J* = 15.9 Hz, H-9a), 3.47 (1H, dd, *J* = 15.9 Hz, H-9b), 4.07 (1H, brs, *J* = 6.2 Hz, H-10), 4.38 (1H, s, H-12a), 4.73 (1H, s, H-12b), 5.73 (1H, d, *J* = 10.0 Hz, H-15), 6.19 (1H, s, H-6), 6.67 (1H, s, H-3'), 6.99 (1H, d, *J* = 10.0 Hz, H-14), 13.45 (1H, s, 5-OH) (see Figures S15–S17 in Supplementary Material).

### Artoindonesianin P (3)

Brown amorphous powder; EIMS, *m*/*z* 368 [M]^+^; HREIMS, *m*/*z* 368.0899 [M + H]^+^ (calcd for C_20_H_16_O_7_ 368.0896); ^1^H NMR (500 MHz, acetone-*d_6_*) *δ* 1.28 (3H, s, H-13), 1.59 (3H, s, H-12), 2.29 (1H, t, *J* = 15.2 Hz, H-9a), 3.11 (1H, dd, *J* = 15.2 Hz, H-9b), 3.34 (1H, dd, *J* = 15.2 Hz, H-10), 6.16 (1H, s, H-6), 6.23 (1H, s, H-3'), 6.39 (1H, s, H-8) (see Figures S18–S20 in Supplementary Material).

### Cycloartobiloxanthone (4)

Yellowish brown amorphous powder; EIMS, *m*/*z* 434 [M]^+^; HREIMS, *m*/*z* 434.1364 [M + H]^+^ (calcd for C_25_H_22_O_7_ 434.1366); ^1^H NMR (500 MHz, acetone-*d_6_*) *δ* 1.41 (3H, s, H-13), 1.51 (3H, s, H-17), 1.53 (3H, s, H-18), 1.74 (3H, s, H-12), 2.41 (1H, t, *J* = 15.5 Hz, H-9a), 3.26 (1H, dd, *J* = 15.2 Hz, H-9b), 3.47 (1H, dd, *J* = 15.2 Hz, H-10), 5.74 (1H, d, *J* = 9.95 Hz, H-15), 6.21 (1H, s, H-6), 6.49 (1H, s, H-3'), 6.99 (1H, d, *J* = 9.95 Hz, H-14) (see Figures S21–S23 in Supplementary Material).

### Artoflavone B (5)

Reddish orange gum. UV (MeOH) *λ*_max_ (log *ɛ*) 205 (4.01), 230 (4.08), 250 (4.08), 260 (4.07), 272 (4.05), 288 (4.06), 298 (4.07), 316 (4.19), 360 (3.61). IR (KBr) 3410, 2975, 1650 cm^−1^. FABMS, *m*/*z* 505 [M + H]^+^; HREIMS, *m*/*z* 505.2216 [M + H]^+^ (calcd for C_30_H_33_O_7_ 505.2148); ^1^H NMR (500 MHz, acetone-*d_6_*) *δ* 1.42 (3H, s, H-16), 1.46 (3H, s, H-13), 1.54 (3H, s, H-22), 1.57 (3H, s, H-12), 1.63 (3H, s, H-21), 1.69 (2H, m, H-17), 2.07 (2H, m, H-18), 3.14 (2H, d, *J* = 7 Hz, H-9), 5.09 (2H, m, H-19 and H-10), 5.61 (1H, d, *J* = 10.1 Hz, H-14a), 6.15 (1H, s, H-6), 6.59 (1H, s, H-3'), 6.61 (1H, d, *J* = 10.1 Hz, H-14), 6.88 (1H, s, H-6'), 13.25 (1H, s, 5-OH). ^13 ^C NMR (125 MHz, acetone-*d_6_*) δ 17.70 (C-22), 17.72 (C-13), 23.39 (C-18), 24.73 (C-9), 25.84 (C-21), 25.90 (C-12), 27.17 (C-16), 42.11 (C-17), 81.35 (C-15), 99.55 (C-6), 101.47 (C-8), 104.77 (C-3'), 105.56 (C-4a), 111.52 (C-1'), 116.00 (C-14), 117.15 (C-6'), 121.69 (C-3), 122.56 (C-10), 124.79 (C-19), 126.93 (C-14a), 132.22 (C-11), 132.36 (C-20), 139.11 (C-5'), 149.72 (C-2'), 149.83 (C-4'), 153.24 (C-8a), 160.35 (C-7), 162.19 (C-2), 162.86 (C-5), 183.30 (C = O, C-4) (see Figures S8, S9, and S14 in Supplementary Material).

### Artonin E (6)

Reddish brown gum. FABMS, *m*/*z* 437 [M + H]^+^; HRFABMS, *m*/*z* 437.1635 [M + H]^+^ (calcd for C_25_H_25_O_7_ 437.1522). ^1^H NMR (500 MHz, acetone-*d_6_*) *δ* 1.41 (6H, s, H-17 and H-18), 1.43 (3H, s, H-13), 1.54 (3H, s, H-12), 3.10 (2H, brd, *J* = 6.99 Hz, H-9), 5.09 (1H, m, H-10), 5.60 (1H, d, *J* = 10.02 Hz, H-15), 6.11 (1H, s, H-6), 6.56 (1H, d, *J* = 9.72 Hz, H-14), 6.59 (1H, s, H-3'), 6.85 (1H, s, H-6') (see Figures S24–S26 in Supplementary Material).

### α-Glucosidase inhibitory activity assay and its kinetics

The inhibitory activity of α-glucosidase (EC3.2.1.20) was carried out with a few changes from literature reported method, using *p*-nitrophenyl-α-D-glucopyranoside at optimal pH of 6.8 (50 mM phosphate buffer)^3^. Inhibitors were dissolved and diluted to a needed concentration in DMSO. Concisely, in 96-well plates to 10 μL of inhibitor or deoxynojirimycin (DNJ) as a control and 40 μL substrate (*p*-NPG, 1.0 mM) in the aforesaid buffer (130 μL) were added 20 μL of the enzyme (0.1 unit/mL). The absorbance of formed *p*-nitrophenol immediately measured with a wavelength of 405 nm at 37 °C. Compound activity was expressed in the concentration when 50% of enzyme activity was inhibited (IC_50_). Calculation of the % of inhibition was as follows:

(1) $$\text{Activity}\;\left(\%\right)=100\left[1+\left(\left[I\right]{/\text{IC}}_{50}\right)\right]$$

Similarly, the enzyme kinetic modes were clarified using a different *p*-nitrophenyl-α-D-glucopyranoside substrate (0, 0.5, 1, and 2 mM) and inhibitors concentrations. Analysis of the data used to determine the individual parameters of curves was prepared in the nonlinear regression program Sigma Plot (SPCC Inc., Chicago, IL, USA). The kinetic parameters, Michaelis–Menten (*K*_m_) and maximum velocity (*V*_max_), were found using Lineweaver–Burk plots. The dissociation constants between inhibitor and enzyme (*K*_i_) were found from Dixon plots. Two inhibition constants for inhibitor binding with either free or enzyme-substrate complex, *K*_I_ or *K*_IS_, were calculated from secondary plots of the slopes of the straight lines or vertical intercept (1/$V_{\max}^{\mathrm{app}}$), respectively, verse the concentration of inhibitors by Equations (2)–(4). *K*_ik_ and *K*_iv_ rate constants were calculated according to Equations (5) and (6) proposed by Yang et al.^20^:

(2) $$1/V=K_{m}/V_{\text{max}}\left(1\;+\;\left[I\right]/K_{I}\right)1/S\;+\;1/V_{\text{ma}x}$$

(3) $$\text{Slope}\;=K_{m}/K_{I}V_{\text{max}}\left[I\right]\;+K_{m}/V_{\text{max}}$$

(4) $$\text{Intercept}\;=\;1/K_{\text{IS}}V_{\text{max}}\left[I\right]\;+K_{m}/V_{\text{ma}x}$$

(5) $$K_{m}=K_{m0}\times \;\left(1\;+\;\left[I\right]/K_{\text{ik}}\right)$$

(6) $$V_{m}=V_{m0}\times \;\left(1\;+\;\left[I\right]/K_{\text{iv}}\right)$$

### Progress curves and time-dependent assay

Time-dependent assays of inhibitors at the different preincubation time (0, 15, 30, 45, 60 and 80 min) were accomplished using 0.05 unit/mL final concentration of α-glucosidase and *p*-nitrophenyl-α-D-glucopyranoside substrate (1 mM) in 50 mM phosphate buffer (pH 6.8) at 37 °C^21^. The progress curves were monitored every 30 s for 30 min. Furthermore, to clearly understand the time-dependent inhibition mode of α-glucosidase, inhibitors were assayed using different concentrations. Accordingly, the progress curves were calculated using Equations (7)–(11). Analysis of the data was prepared by the nonlinear regression program Sigma Plot (SPCC Inc., Chicago, IL, USA):

(7) $$\frac{{\left[P\right]}_{t}}{\left[E\right]}=\;v_{s}t+\;\frac{v_{i}-v_{s}}{k_{\text{obs}}}(1-e^{{-k}_{\text{obs}}t})$$

where [P]*_t_* is the concentration of product formed and [E] is the total enzyme concentration.

(8) $$v/v_{0}=\;\text{exp}\;\left(-k_{\text{obs}}t\right)$$

(9) $$\text{Enz}+I\overset{k_{3}}{\underset{k_{4}}{⇄}}\text{Enz}-I$$

(10) $$k_{\text{obs}}=k_{4}\left(1+\left[I\right]/K_{i}^{\text{app}}\right)$$

(11) $$K_{i}^{\text{app}}=k_{4}/k_{3}$$

### Binding affinity measurement

180 µL of 50 mM phosphate buffer (pH 6.8) with 10 µL of 0.2 unit/mL α-glucosidase were added into the 96-well black immuno-plates then 10 µL different concentrations (2–32 µM) of inhibitor were added^22^. Fluorescent emission spectra were recorded from 300 to 400 nm, emission slits adjusted to 2.0 nm, and the excitation was 250 nm, using spectrophotometer (SpectraMax M3). There was no significant emission from any ingredient in the assay system under given experimental conditions (i.e. emission from 300 to 400 nm). A level of affinity was expressed with *K*_SV_, which was analysed using the Stern–Volmer equation:

(12) $$F_{0}-F=1+K_{\text{SV}}\left[Q_{f}\right]$$

where *F*_0_ and *F* are the fluorescence intensities in the absence and presence of a quencher. Q_f_ is a concentration of compounds.

### Molecular docking calculation

To predict the binding conformation of competitive inhibitors to *Saccharomyces cerevisiae* α-glucosidase, molecular docking studies were performed by GOLD Suite 5.2.2 (the Cambridge Crystallographic Data Centre, UK). The three dimensional (3D) structure of *Saccharomyces cerevisiae* α-glucosidase had been built in the previous study^23^. The 3D structures of compounds were prepared using the sketching tool and their geometries were optimised by Minimisation protocol in Discovery Studio (DS) 2018 (BIOVIA, San Diego, CA, USA). The smart minimiser algorithm was applied with CHARMm force field. The environment of the system was set an implicit solvent using Born molecular volume (GBMV). A docking site was defined within 20 Å from the centre of the mass on M69, H111, F157, R213, D214, and R312, which are conserved residues in the active site. Each compound was docked 100 times using the genetic algorithms (GA) with the default parameters^24^. The best binding conformation for each compound was selected based on the GOLD fitness score from the most populated cluster in each compound.

### Statistical analysis

All experiments were made at least thrice and analysed using Sigma Plot version 10.0. A value of *p* < 0.05 was considered to be a significant difference.

## Results and discussion

### Isolation of α-glucosidase inhibitors

**Figure 1.**
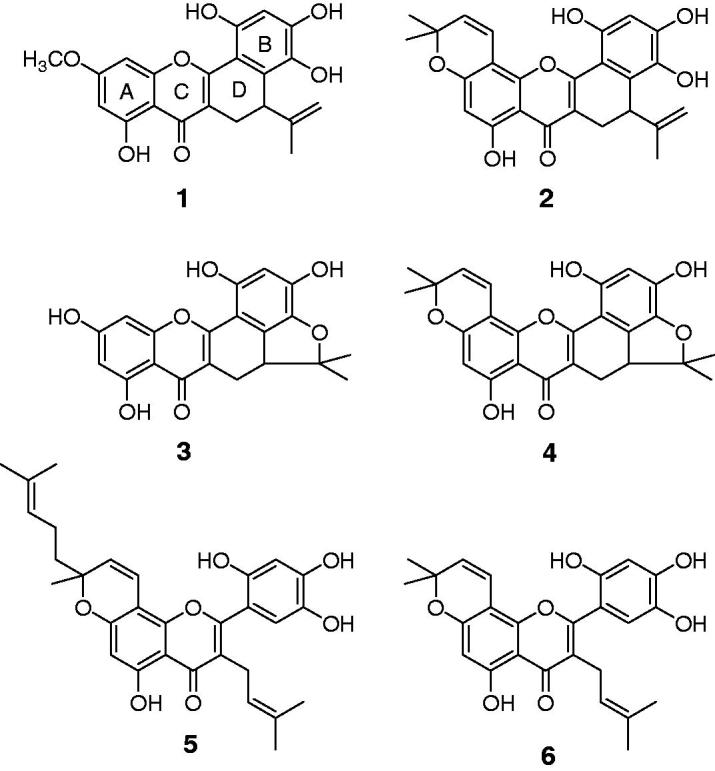
Chemical structures of compounds **1**–**6** from the barks of *Artocarpus elasticus*.

First, from the methanol extract of *A. elasticus* barks, we purified six compounds (**1**–**6**) displaying α-glucosidase inhibition. As shown in Figure 1, compounds (**2**–**4**, and **6**) were identified as artobiloxanthone (**2**), artoindonesianin P (**3**), cycloartobiloxanthone (**4**), and artonin E (**6**) by our spectroscopic data (see Supplementary Material), compared with the previously reported^13^^,^^17^. Compounds **1** and **5** emerged to be new compounds named as artoindonesianin W (**1**) and artoflavone B (**5**).

Compound **1** having molecular formula C_21_H_18_O_9_ with 13 degrees of unsaturation was established on basis of HRFABMS data [M + H]^+^ (*m/z* 383.1149, calcd 383.1053). The extra 4 degrees of unsaturation after counting double bonds were deduced from the tetracyclic skeleton of compound **1**. The hydrogen bonding hydroxyl group (C5-OH, *δ*_H_ 13.21) and α, β-unsaturated carbonyl (*δ*_C_ 181.1) were consistent with a feature of a flavone structure. ^1^H and ^13 ^C NMR data in conjunction with DEPT experiments indicated the presence of 21 carbon atoms, consisting of the following functional groups: 1 methylene (sp^2^), 1 methylene (sp^3^), 1 methine (sp^3^), 3 methines (sp^2^), 2 methyls and 13 quaternary carbons. Allylic coupling between H6 (*δ*_H_ 6.29, d) and H8 (*δ*_H_ 6.65, d) confirmed meta substituents of ring A. The C7-OCH_3_ was proved by a strong HMBC of OCH_3_ (*δ*_H_ 3.89) with C7 (*δ*_H_ 166.1). The ring B with five substituents was deduced by HMBC of H3' (*δ*_H_ 6.50, s) with C2' (*δ*_C_ 151.4) and C4' (*δ*_C_ 151.2). The presence of ring D with propenyl group was deduced from proton coupling networks across H9_a/b_/H10/H12/H13 in the COSY spectrum. The propenyl moiety was confirmed by a strong HMBC of exo-methylene H13a/b and C12-CH_3_ (*δ*_C_ 22.0). The location of propenyl moiety on C10 was confirmed by HMBC of H10 (*δ*_H_ 3.99) and C11 (*δ*_C_ 145.4) (Figures S3–S6 in Supplementary Material and Table S1 in Supplementary Material). Thus, compound **1** was determined to be 1,3,4,8-tetrahydroxy-10-methoxy-5-(prop-1-en-2-yl)-5,6-dihydro-benzo[c]xanthen-7-one, named as artoindonesianin W.

Compound **5** had the molecular formula C_30_H_32_O_7_ made on basis of HRFABMS data with [M + H]^+^ ion at 505.2216 (calcd 505.2148). It has a feature of the flavone skeleton with hydrogen bonding C5-OH (*δ*_H_ 13.2) and α, β-unsaturated carbonyl (*δ*_C_ 183.0). The ring A having five substituents was confirmed a singlet H6 (*δ*_H_ 6.15, s) which has HMBC correlation with C5 (*δ*_C_ 162.9) and C7 (*δ*_C_ 160.4). Two singlet protons of H3' (*δ*_H_ 6.59) and H6' (*δ*_H_ 6.88) showed four substituents of ring B. The positions of H3' and H6' were confirmed by HMBC correlation of H3' with C2' (*δ*_C_ 149.7) and C4' (*δ*_C_ 149.8), and H6' with C1' (*δ*_C_ 111.5) and C5' (*δ*_C_ 139.1). The prenyl group was confirmed by a typical coupling network across H9/H10/H12/H13 in the COSY spectrum. The position of a group was confirmed by HMBC of H9 (*δ*_H_ 3.14) with C3 (*δ*_C_ 121.7), and carbonyl C4 (*δ*_C_ 183.0). The presence of pyran moiety was deduced from proton coupling of H14 (*δ*_H_ 5.61, d) with H14a (*δ*_H_ 5.61, d) and HMBC between H14a and oxygenated carbon C15 (*δ*_C_ 81.4). The proton coupling network across H17/H18/H19/H21/H22 indicated the presence of the 2-methyl-2-pentenyl group. The locations of 2-methyl-2-pentenyl and methyl groups were confirmed by HMBC correlations of both H16 (*δ*_H_ 1.42) and H17 (*δ*_H_ 1.69) with C15 (Figures S10–S13 in Supplementary Material and Table S1 in Supplementary Material). Thus compound **5** was determined to be (-)-2-(2,4,5-trihydroxyphenyl)-3-(3-methyl-2-buten-1-yl)-5-hydroxy-8-methyl-8-(4-methyl-3-penten-1-yl)-4H,8H-benzo[1,2-b:3,4-b']dipyran-4-one, named as artoflavone B.

### α-Glucosidase inhibition

The known flavonoids (**2**–**4** and **6**) are reported as effective antioxidant and anticancer^17^^,^^25^. This study tried to find out a new biological function of the isolated compounds (**1**–**6**) based on α-glucosidase inhibition. α-Glucosidase inhibitory activity was screened at different concentrations using the modified UV assay^3^. All six compounds (**1**–**6**) showed a significant inhibition towards α-glucosidase with IC_50_s ranging between 7.6 and 25.4 μM (Table 1). The inhibitory potencies varied with the modification of ring B. Dihydrobenzoxanthones (**1**–**4**) were formed by cyclisation of isoprenyl group on C-3 with the C-6′ at ring B of prenylated flavone. Dihydrobenzoxanthone **2** (IC_50_ = 8.6 μM) is twice more effective than its mother compound **6** (IC_50_ = 16.2 μM). Both dihydrobenzoxanthone **2** and furanodihydrobenzoxanthone **4** (IC_50_ = 9.6 μM) showed similar inhibitory potential.

**Table 1.** Inhibitory effects of compounds **1**–**6** on α-glucosidase.

Compounds	α-Glucosidase			
	IC_50_ (μM)^a^	Kinetic mode (*K*_i_, μM)^b^	*K*_I_ (μM)	*K*_IS_ (μM)
**1**	7.6 ± 0.2	Competitive (2.9 ± 0.4)	NT^c^	NT
**2**	8.6 ± 0.5	Competitive (5.1 ± 0.3)	NT	NT
**3**	25.4 ± 0.7	Competitive (12.6 ± 0.7)	NT	NT
**4**	9.6 ± 0.6	Competitive (5.8 ± 0.4)	NT	NT
**5**	8.8 ± 0.3	Mixed type I (5.4 ± 0.7)	5.4	20.4
**6**	16.2 ± 0.8	Mixed type I (15.8 ± 0.9)	15.4	51.7
DNJ^d^	42.5 ± 0.9	NT	NT	NT

a Sample concentration which led to 50% enzyme activity loss.

b *K*_i_ is the inhibition constant.

c NT is not tested.

d Deoxynojirimycin (DNJ) is used as a positive control.

**Figure 2.**
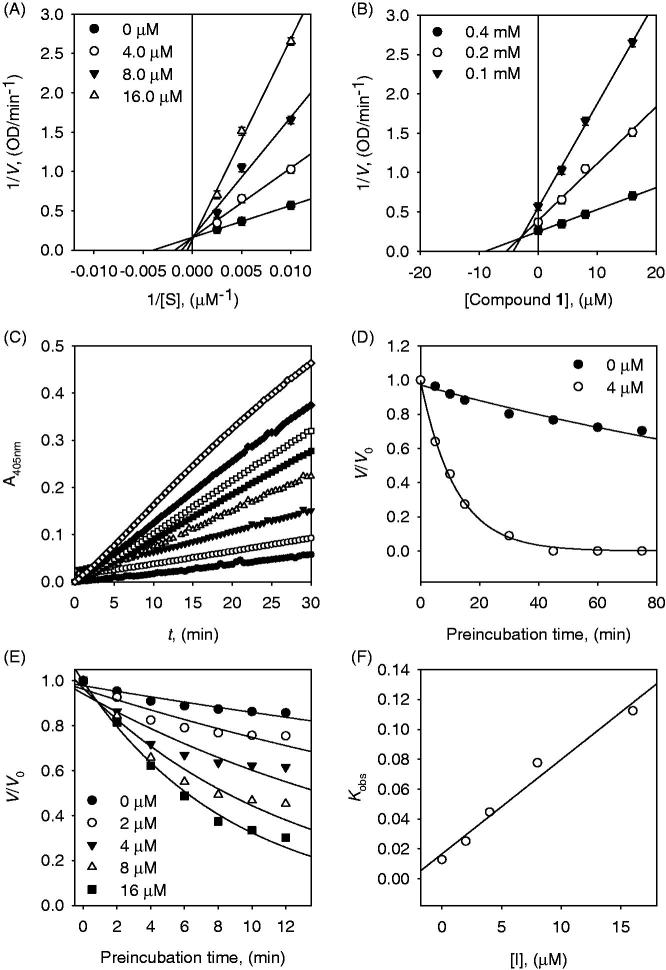
(A) Lineweaver–Burk and (B) Dixon plots for the inhibition of compound **1** on the α-glucosidase activity. (C) Slow-binding inhibition at different preincubation time (◇: 0; ◆: 5; □: 10; ■: 15; △: 30; ▼: 45; ○: 60; ●: 75 min) for compound **1** at 4 μM. (D) Inhibition as a function of preincubation time for compound **1**. (E) Time course of the inactivation of α-glucosidase compound **1**. (F) *k*_obs_ on dependence on different concentrations of compound **1**.

**Figure 3.**
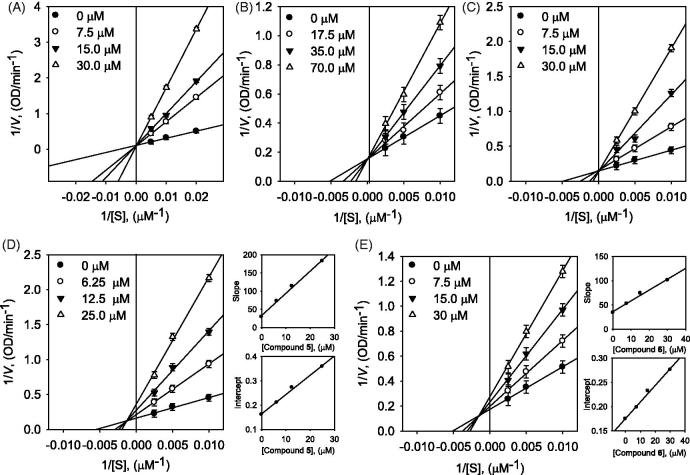
Lineweaver–Burk plots for α-glucosidase (A) compound **2**, (B) compound **3**, (C) compound **4**, (D) compound **5**, and (E) compound **6**. Insets represent the secondary plots of the slope and intercept of the straight lines vs. concentrations of compounds.

**Figure 4.**
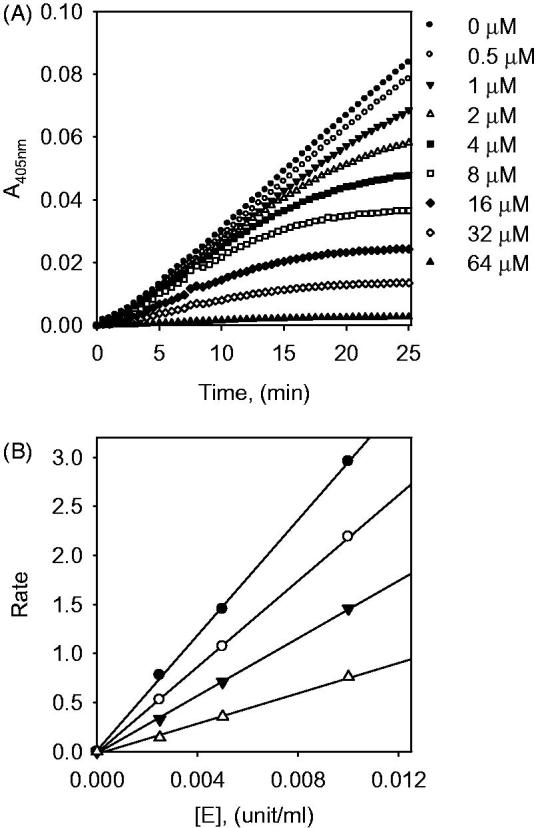
(A) Dose-dependent effect of compound **1** on α-glucosidase inhibition. (B) The catalytic activity of α-glucosidase as a function of enzyme concentration at different concentrations of compound **1**.

To investigate the inhibition mechanism, we conducted various kinds of evaluations such as dose-dependence, reversibility, Lineweaver–Burk plots, Dixon plots, Yang’s method and the time dependence of inhibition of α-glucosidase by inhibitors (Figures 2, 3 and 4). All tested compounds showed dose-dependence inhibition of an enzyme (Figure 4(A)). They all successively demonstrated the relationship between enzyme activity and concentrations. The α-glucosidase inhibition by representative inhibitor **1** (IC_50_ = 7.6 μM) demonstrated in Figure 4(B), which was obtained by the plotting of remaining enzyme activity vs. the concentration of enzyme at different inhibitor concentrations. Increasing of the inhibitor **1** concentrations provided a reduction of the slopes of lines. An indication of compound **1** as a reversible inhibitor was concluded from that the family of straight lines was passed through the origin.

First of all, dihydrobenzoxanthones (**1**–**4**) showed competitive inhibition behaviour. Competitive inhibitors of α-glucosidase have rarely been reported from natural phenolic compounds and structural features have not been systematically investigated. A detailed kinetic analysis of the inhibition was modelled using double reciprocal plots. This analysis estimated no change of *V*_max_ and the increase of *K*_m_ as expected to competitive inhibition. It can be seen directly from Figures 2(A) and 4(A–C), families of 1/*V* vs. 1/[S] regression line have the common intercept on the y-axis. To further confirmation of competitive inhibition mode, the results were applied to Yang’s method^20^. The new kinetic parameter *K*_ik_ can be fit to Equation (5), while *K*_iv_ can be fit to Equation (6), from plotting *K*_m_ and *V*_max_ against inhibitor concentrations. The *K*_ik_/*K*_iv_ ratios were calculated between 29.8 and 55.8 as expected for competitive inhibition behaviour (Table 2 and Table S2 in Supplementary Material).

**Table 2.** Determination of *K*_ik_/*K*_iv_ ratios by α-glucosidase enzyme inhibitory behaviours.

Compounds	[I] (μM)	*V*_max_	*K*_m_	*K*_ik_/*K*_iv_
**1**	0	6.17	250.44	–
	4.0	6.30	558.66	55.80
	8.0	6.50	915.24	48.88
	16.0	7.55	1929.38	29.79
**5**	0	6.20	182.35	–
	7.5	4.74	344.65	3.79
	15.0	3.67	418.39	3.18
	30.0	2.79	508.39	3.25

Inhibitor **1** displayed to be a slow-binding inhibitor because the residual activity of the enzyme was decreased as a function of preincubation time (Figure 2(C)). The time dependence of the hydrolysis rate by α-glucosidase was evaluated by measuring of the enzyme residual activity at different concentrations of inhibitor **1** (0, 5, 10, 20, and 40 μM) over different time points. The results were fitted to (7) and (8) to determine *k*_obs._ Figure 2(F) represents the relationship between *k*_obs_ and [I] to determine the kinetic profile of inhibitor **1** (simple reversible slow-binding enzyme isomerisation or mechanism-based inhibition). The data for inhibitor **1** were fit to the slow binding Equation (7) illustrating no deviation from linearity of *k*_obs_ on the concentration of α-glucosidase. This mechanism can be shaped to the simple reversible slow-binding model. By fitting Equations (10) and (11) we established the parameters of *k*_3_ = 0.0437 µM^−1 ^min^−1^, *k*_4_ = 0.0166 min^−1^, and $K_{i}^{\mathrm{app}}$= 0.3795 µM for inhibitor **1**.

As shown in Figure 3(D), the common intercept of Lineweaver–Burk plots was on the left of the vertical axis and above the horizontal axis determined the type of inhibition for inhibitor **5** as mixed. Mixed type inhibitor may have different affinity for the substrate bound (mixed type I) and free enzyme (mixed type II). The results of compound **5** were applied to Equations (3) and (4) to calculate *K*_I_ and *K*_IS_ from secondary plots of *K*_m_/*V*_max_ and 1/*V*_max_ vs. concentration of compound **5**. The compound **5** was found to be mixed type I (*K*_I_ = 5.4 μM < *K*_IS_ =20.4 μM). The *K*_i_ value of compound **5** was determined to be 5.4 μM by Dixon plots (Figure S27 in Supplementary Material).

### Binding affinities between α-glucosidase and compounds

**Figure 5.**
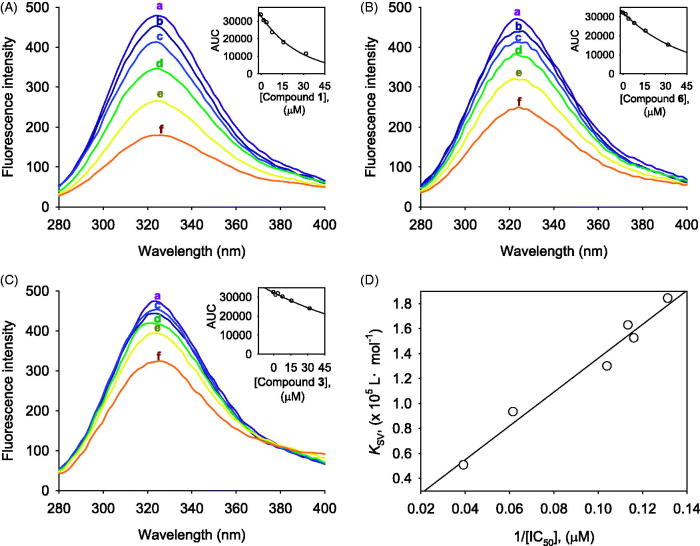
The fluorescence spectra (A–C) of α-glucosidase at different concentrations (0, 2, 4, 8, 16, and 32 μM for curves a→f, pH 6.8, *T* = 37 °C), (A) For compound **1**, (B) For compound **6**, (C) For compound **3. **(D) The correlation between inhibitory potencies (IC_50_s) and Stern–Volmer constant (*K*_SV_).

α-Glucosidase has many fluorophore residues of 20 Trp, 26 Tyr, and 41 Phe (Figure S29 in Supplementary Material), of which the intrinsic fluorescence might be changed by the function of inhibitor affinity^22^. Thus, α-glucosidase is proper enzyme to be estimated the affinity with inhibitor by using the change of fluorescence intensity called as fluorescence quenching. This study tried to measure enzyme affinity of inhibitors by fluorescence quenching method. Figure 5(A–C) is fluorescence spectra of compounds **1**, **6**, and **3** (see Figure S28 in Supplementary Material for compounds **2**, **4**, **5**). The dose-dependent lowering of the fluorescence intensity was observed on the increase of the concentrations of inhibitors. Importantly, the decreasing tendency of fluorescence quenching was highly correlated with inhibitory potencies (IC_50_) (Figure 5(D)). For example, the inhibitory potencies (**1 **>** 6** > **3**) could be ranked in order of affinity levels (*K*_SV_) of inhibitors as follows: **1** (*K*_SV_ = 1.84 × 10^5^Lmol^−1^) > **6** (*K*_SV_ = 0.93 × 10^5^Lmol^−1^) > **3** (*K*_SV_ = 0.51 × 10^5^Lmol^−1^), as presented in Table 3.

**Table 3.** Fluorescence quenching effect of compounds **1**–**6** on α-glucosidase.

Compounds	α-Glucosidase				
	*K*_SV_ (×10^5^ L·mol^–1^)	*R*^a^	*K*_A_ (×10^6^ L·mol^–1^)	*n*	*R*^b^
**1**	1.84	1.00	0.79	1.18	0.99
**2**	1.53	0.98	0.76	1.10	0.99
**3**	0.51	1.00	0.57	0.81	1.00
**4**	1.29	1.00	0.79	1.02	0.98
**5**	1.63	1.00	0.81	1.24	0.99
**6**	0.93	0.97	0.78	1.02	0.98

a *R* is the correlation coefficient for the *K*_SV_ values.

b *R* is the correlation coefficient for the *K*_A_ values.

### Molecular docking calculation

**Figure 6.**
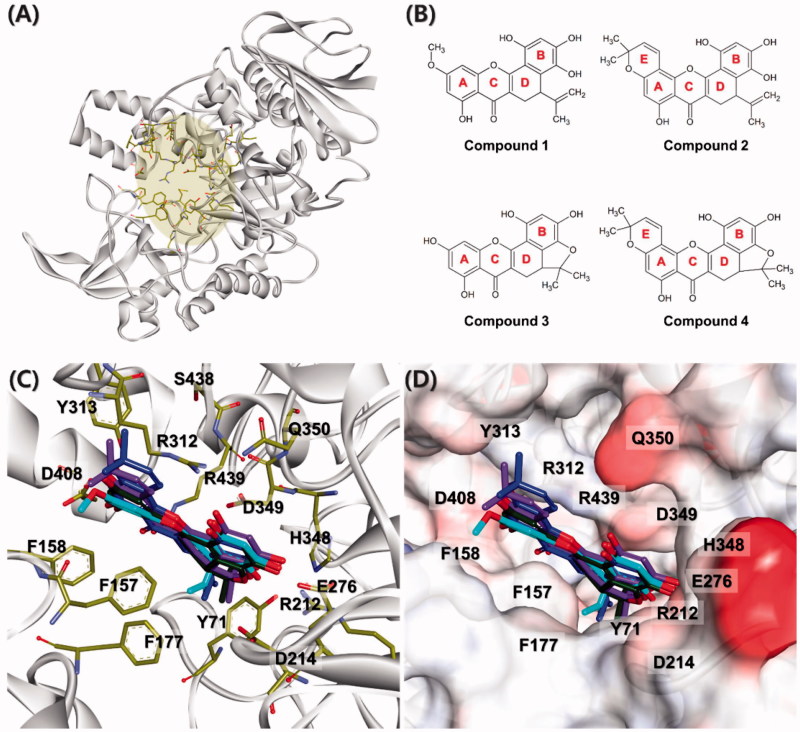
Structural information for dock conformation of competitive inhibitors to the *Saccharomyces cerevisiae* α-glucosidase. (A) The homology model structure of the *Saccharomyces cerevisiae* α-glucosidase. The active site is shown as the yellow region and key residues of the active site represented by yellow stick models. (B) Chemical structures of compounds **1**–**4**. (C and D) The superposition of docked conformations with compounds **1**–**4** in the active site. Compounds **1**–**4** are represented as cyan, blue, green, and purple stick models, respectively.

**Figure 7.**
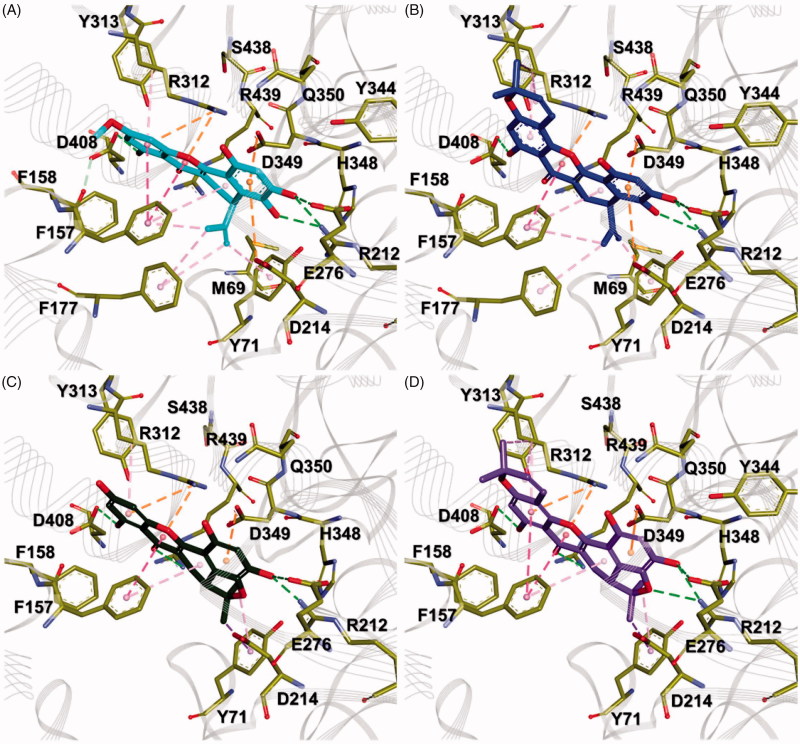
Comparison of binding conformation between each compound. (A–D) Compounds **1**–**4** are shown as cyan, blue, green, and purple thick stick models, respectively. Key residues in the active site are represented by yellow stick models. Hydrogen bond, π–π, π–σ, and π–cation (anion) interactions are shown as light green, hot pink, pink, and orange dashed lines, respectively.

In order to predict the proper binding conformations of the compounds **1**–**4**, molecular docking calculations were performed into the *Saccharomyces cerevisiae* α-glucosidase (Figure 6(A and B)). The docking results revealed that all compounds have similar binding conformations (Figure 6(C,D)). The B and D rings of all compounds interacted the D214 and E276 known as key residues of α-glucosidase^26^^,^^27^, while A, C, and E rings interacted with the residues which were relatively more exposed to solvent than other residues in the binding pocket (Figure 6(D)). The B rings of compounds **1** and **2** were found to interact with D214 and D349 by π-anion interactions, as well as with M69, Y71, S438, R439, Y344, and H348 by van der Waals (vdW) interactions (Figure 7(A,B)). Two hydroxyl groups of the B ring formed strong bidentate hydrogen bonds with the side chains of R212 and E276. On the other hand, in compounds **3** and **4**, these interactions were observed by a hydroxyl and an ether groups (Figure 7(C,D)). The D rings of all compounds contacted with the side chain of Y71 and F157 by π–alkyl interactions, as well as vdW interactions with M69, D214, D349, and R439. In the case of compounds **1** and **2**, an isobutylene group formed π–alkyl interactions with F177. The A and C rings of all compounds were stabilised by π–π interactions with the phenyl group of F157. These rings were found to interact with R312 by π–cation and π–alkyl interactions. The hydroxyl group of the A ring formed hydrogen bond with D408. Moreover, it also found the vdW interactions, which were observed between the methoxy group of the A ring and F157, F158, and Y313. E rings of compounds **2** and **4** involved π–alkyl interactions with the side chain of R312 as shown in Figure 7(B,D). The dimethyl group of E ring also interacted with R312 by π–alkyl interactions and vdW interactions were found with Y313 and D408.

Consequently, the binding of all compounds seems to be stabilised by π–π interactions with F157 and π–alkyl interactions with R312. It is consistent with the fact that hydrophobic interactions, including π–π and π–alkyl interactions, between inhibitors and α-glucosidase, might play a significant role in inhibitory activities^28^^,^^29^. However, structural analysis of compound **3** indicated that the interactions with F157 and R312 are insufficient compared to other compounds because it had a hydroxyl group in the A ring. On the contrary, compound **1** had a methoxy group in the A ring. In particular, compounds **2** and **4** contained additionally dimethyl groups in the E ring. These functional groups increase the hydrophobicity of the compounds, thereby enhancing hydrophobic interactions with F157 and R312. In these structural differences, compound **3** would lead to having a lower inhibitory activity than other compounds. Our molecular docking results of compounds **1**–**4** into the *Saccharomyces cerevisiae* α-glucosidase will provide structural insights into the development of novel competitive inhibitors.

## Conclusions

The six α-glucosidase inhibitors (**1**–**6**), including two new compounds, were isolated from the barks of *A. elasticus*. All isolated compounds showed a significant inhibition with IC_50_s of 7.6–25.4 μM. A full set of kinetic study has been completed for dihydrobenzoxanthones (**1**–**4**) to be competitive and reversible simple slow-binding inhibitors. Competitive inhibition of α-glucosidase has rarely been reported from natural phenolic compounds. Binding affinities and specific binding sites were also confirmed by fluorescence quenching and molecular modelling study. Molecular modelling study showed that all inhibitors have sufficient hydrophobic interaction with F157 and R312 in the active site. These results led a dihydrobenzoxanthone to be a lead skeleton of a competitive inhibitor toward α-glucosidase.

## Supplementary Material

Supplemental Material
