# Supplementary material for: Competitive α-glucosidase inhibitors, dihydrobenzoxanthones, from the barks of Artocarpus elasticus
Source: J Enzyme Inhib Med Chem. 2019 Sep 3;34(1):1623–32. doi: 10.1080/14756366.2019.1660653 (PMC6735331; doi:10.1080/14756366.2019.1660653)
Supplement: Supplemental Material [file IENZ_A_1660653_SM1667.pdf]

# Supplementary information

**$^{13}$C NMR spectroscopic data of compounds:**

***Artobiloxanthone (2)*** $^{13}$C NMR (125 MHz, acetone-$d_6$) δ 21.99 (C-12), 22.27 (C-9), 28.20 (C-18), 28.54 (C-17), 38.14 (C-10), 78.71 (C-16), 99.78 (C-6), 102.03 (C-8), 103.7 (C-3'), 105.51 (C-4a), 107.03 (C-1'), 111.43 (C-3), 111.91 (C-13), 116.14 (C-14), 127.88 (C-6'), 129.88 (C-15), 136.68 (C-5'), 145.35 (C-4'), 150.92 (C-11), 151.57 (C-2'), 152.31 (C-8a), 159.48 (C-7), 161.80 (C-5), 162.57 (C-2), 181.27 (C=O, C-4).

***Artoindonesianin P (3)*** $^{13}$C NMR (125 MHz, acetone-$d_6$) δ 21.18 (C-9), 23.07 (C-12), 28.54 (C-13), 50.05 (C-10), 94.44 (C-11), 95.23 (C-8), 100.11 (C-6), 105.22 (C-1'), 105.27 (C-4a), 103.73(C-3'), 112.96 (C-3), 133.92 (C-6'), 138.35 (C-5'), 147.88 (C-4'), 152.16 (C-2'), 158.57 (C-8a), 162.63 (C-5), 163.16 (C-2), 165.39 (C-7), 181.98 (C=O, C-4).

***Cycloartobiloxanthone (4)*** $^{13}$C NMR (125 MHz, acetone-$d_6$) δ 20.61 (C-9), 23.10 (C-13), 28.51 (C-18), 28.53 (C-17), 28.71(C-12), 47.78 (C-10), 78.99 (C-16), 93.97 (C-11), 100.19 (C-6), 102.14 (C-8), 105.13 (C-4a), 105.64 (C-1'), 105.64 (C-3'), 112.95 (C-3), 116.21 (C-14), 128.16 (C-15), 133.96 (C-6'), 138.17 (C-5'), 147.30 (C-4'), 151.84 (C-2'), 152.31 (C-8a), 159.77 (C-5), 161.78 (C-2), 162.88 (C-7), 181.69 (C=O, C-4).

***Artonin E (6)*** $^{13}$C NMR (125 MHz, acetone-$d_6$) δ 18.02 (C-13), 25.03 (C-9), 26.19 (C-12), 28.62 (C-17 and C-18), 79.12 (C-16), 100.06 (C-6), 101.94 (C-8), 105.07(C-3'), 105.94 (C-4a), 111.76 (C-1'), 115.79 (C-14), 117.40 (C-6'), 121.99 (C-3), 122.84 (C-10), 128.36 (C-15), 132.67 (C-11), 139.45 (C-5'), 149.83 (C-4'), 150.13 (C-2'), 153.61 (C-8a), 160.29 (C-7), 162.59 (C-5), 163.09 (C-2), 183.63 (C=O, C-4).

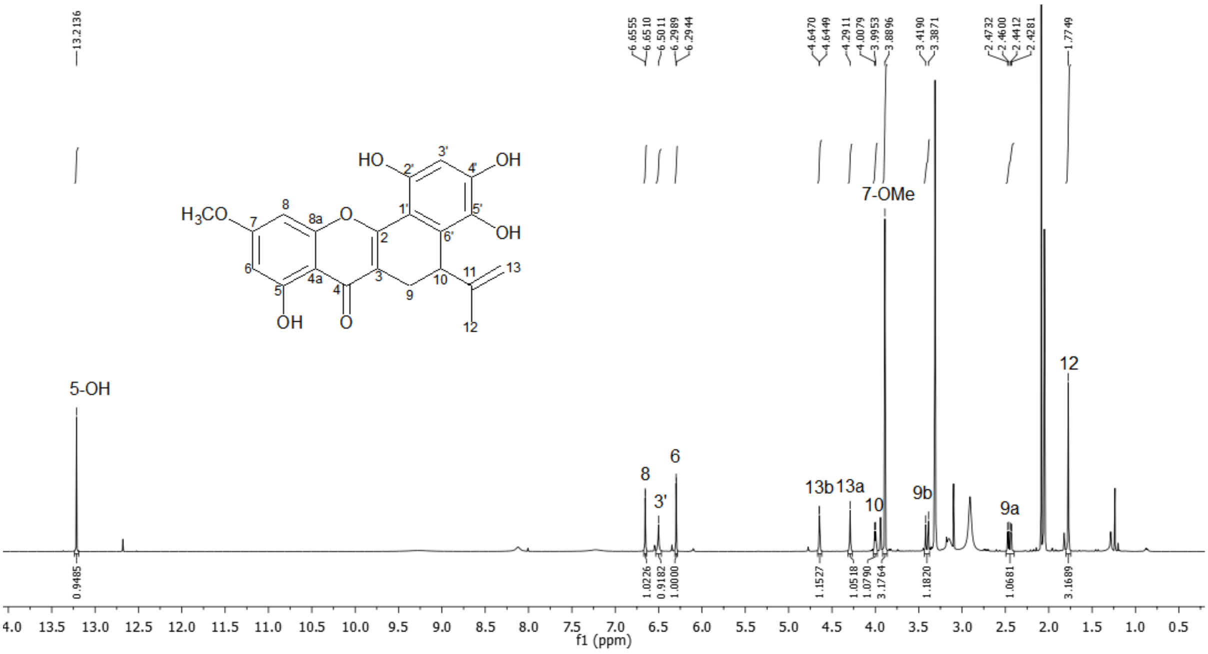

**Figure S1.** $^{1}$H-NMR spectrum of compound **1** (500mHz, acetone-$d_6$)

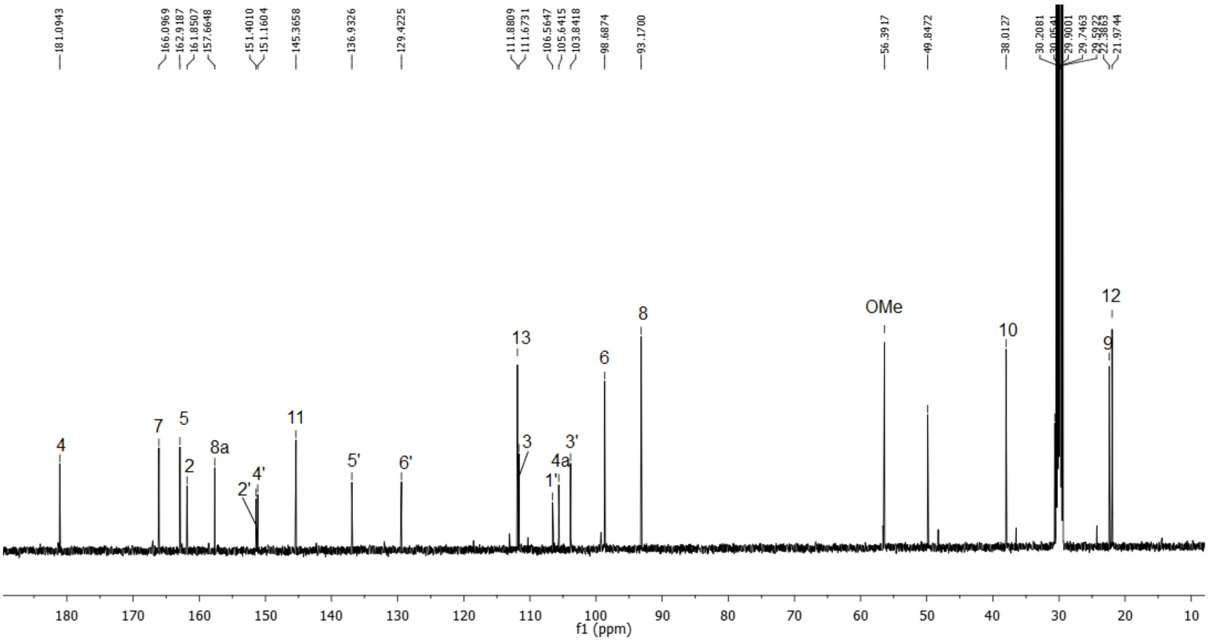

**Figure S2.** $^{13}$C-NMR spectrum of compound **1** (125mHz, acetone-$d_6$)

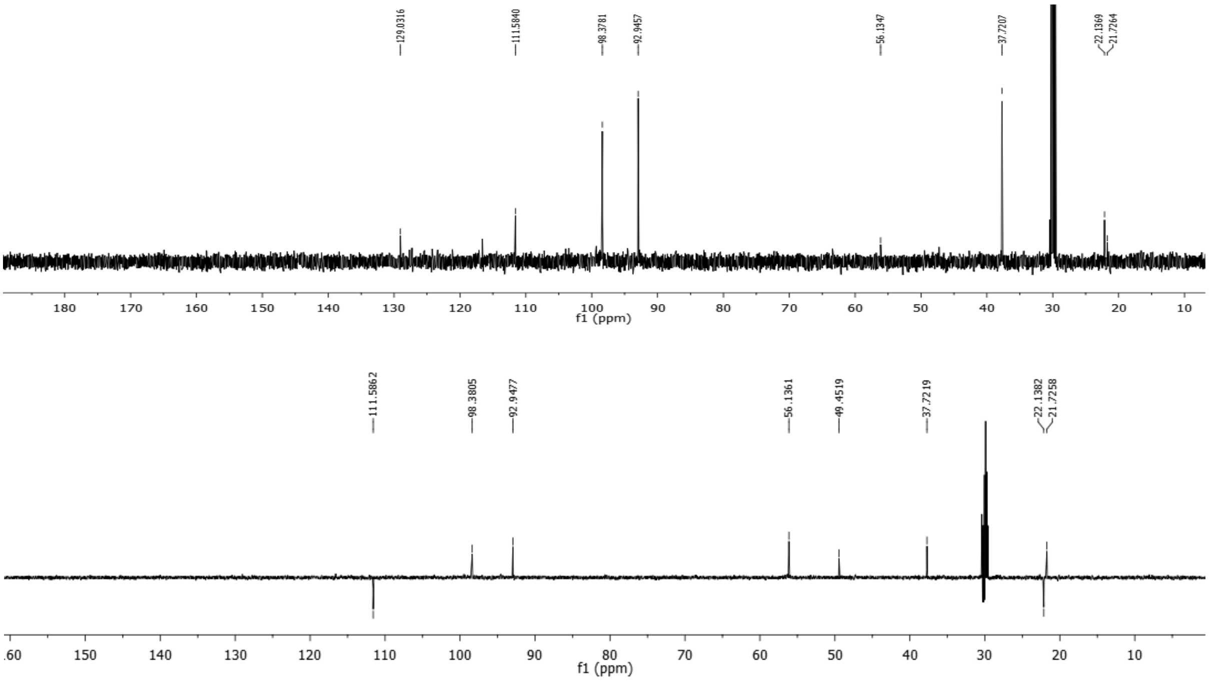


**Figure S3.** DEPT-90 and -135 spectrum of compound **1**.

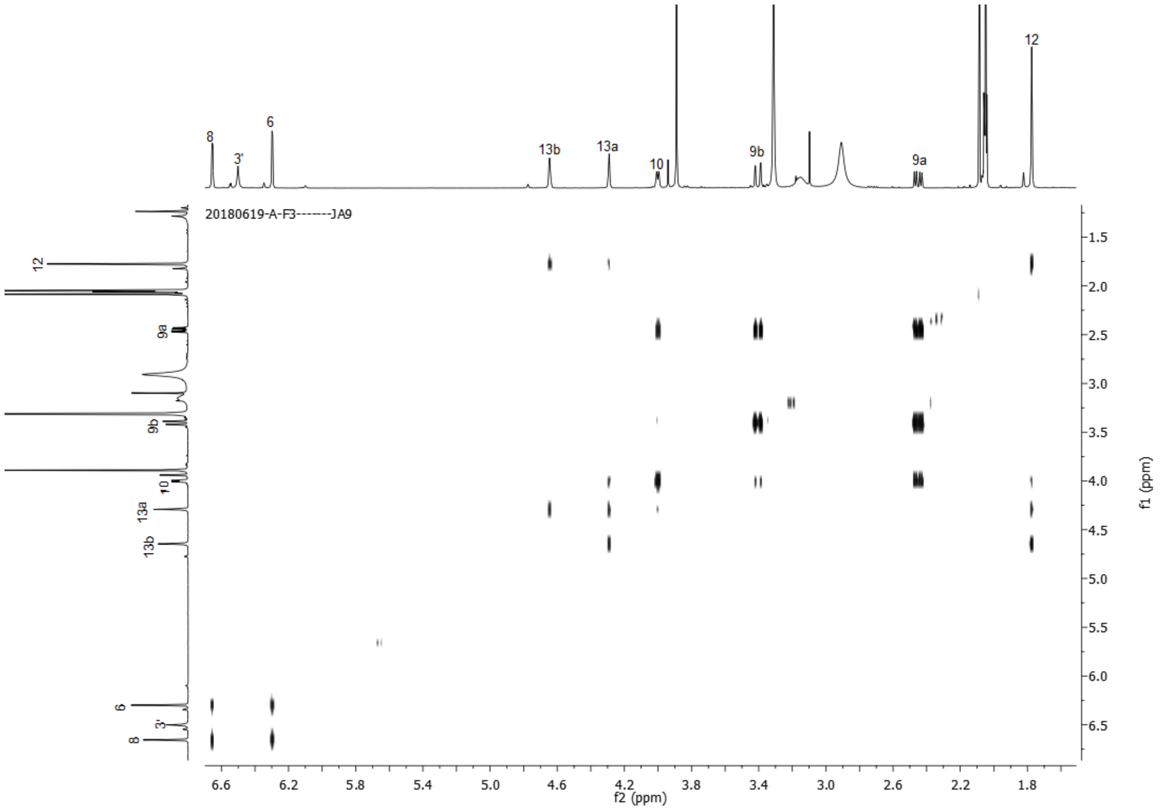


**Figure S4.** COSY spectrum of compound **1**.

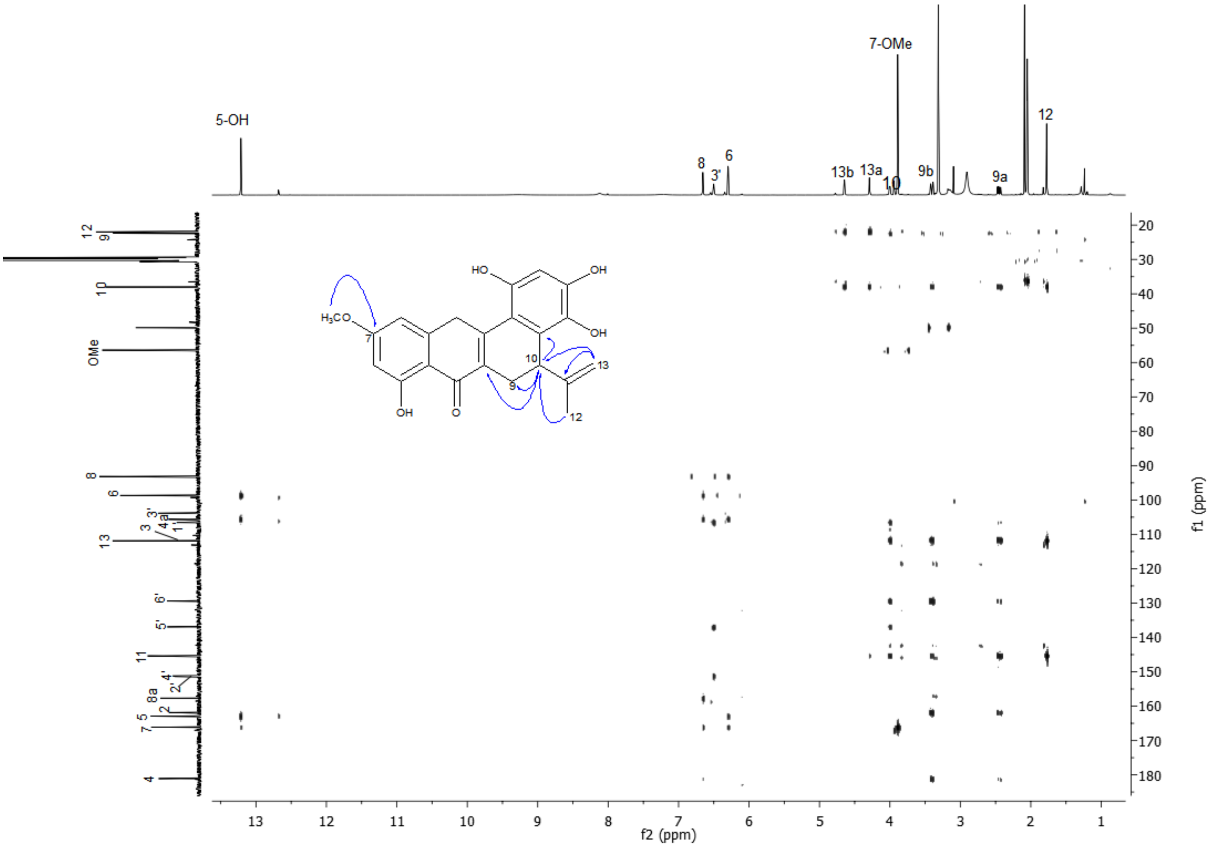


**Figure S5.** HMBC spectrum of compound **1**.

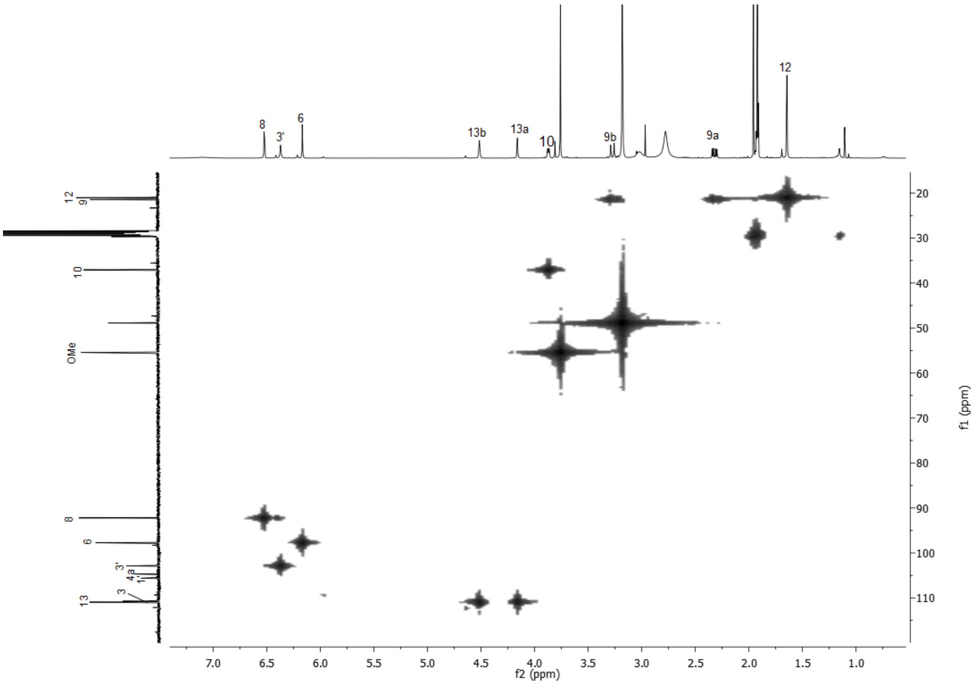


**Figure S6.** HMQC spectrum of compound **1**.

```
[ Mass Spectrum ]
Data  : F3-1                    Date : 21-Dec-2018 16:54
Sample: -
Note : -
Inlet : Direct                  Ion Mode : FAB+
Spectrum Type : Normal Ion [MF-Linear]
RT : 0.45 min         Scan# : 4
BP : m/z 154.0829     Int.  : 237.67
Output m/z range : 100.0000 to 1002.0772     Cut Level : 0.00 %
```

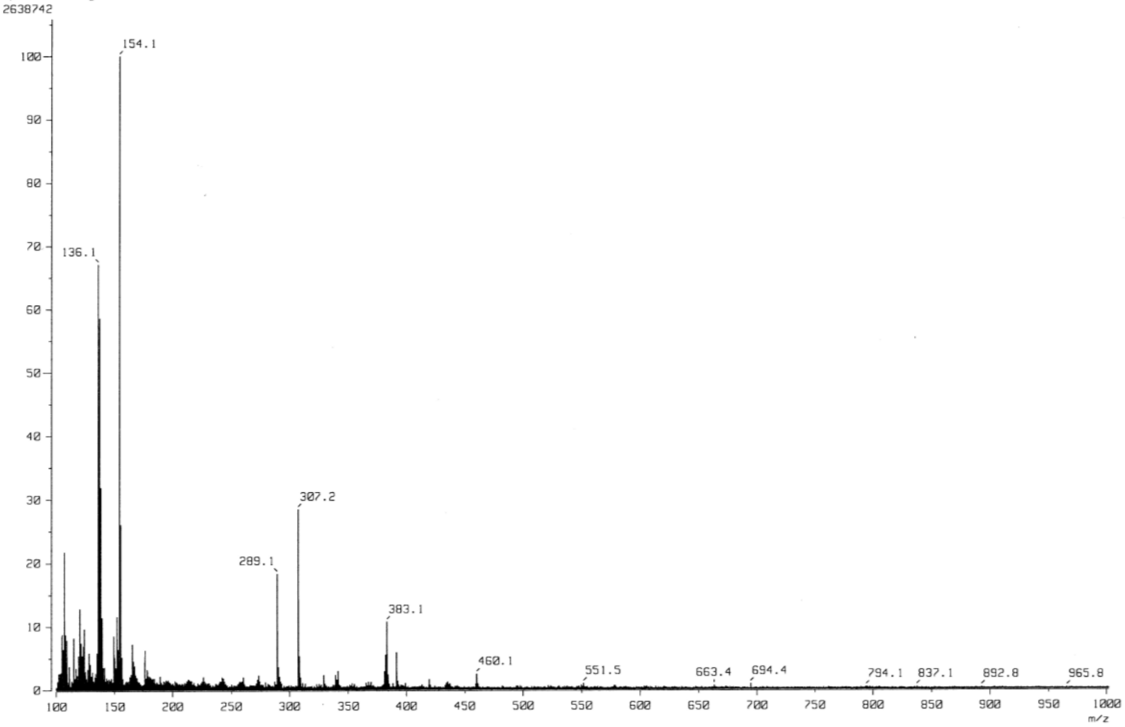


```
[ Elemental Composition ]                                Pag
Data   : A-F3_HR                 Date : 10-Jan-2019 16:29
Sample: -
Note : -
Inlet : Direct                   Ion Mode : FAB+
RT : 4.67 min                    Scan#: 57
Elements : C 110/1, H 130/1, O 8/1
Mass Tolerance      : 100ppm, 3mmu if m/z > 30
Unsaturation (U.S.) : 0.0 - 30.0

Observed m/z Int%   Err[ppm / mmu]    U.S.  Composition
  383.1149   100.0     +4.8 /  +1.9   12.5  C 21 H 19 O 7
```

**Figure S7.** FABMS and HRFABMS data of compound **1**.

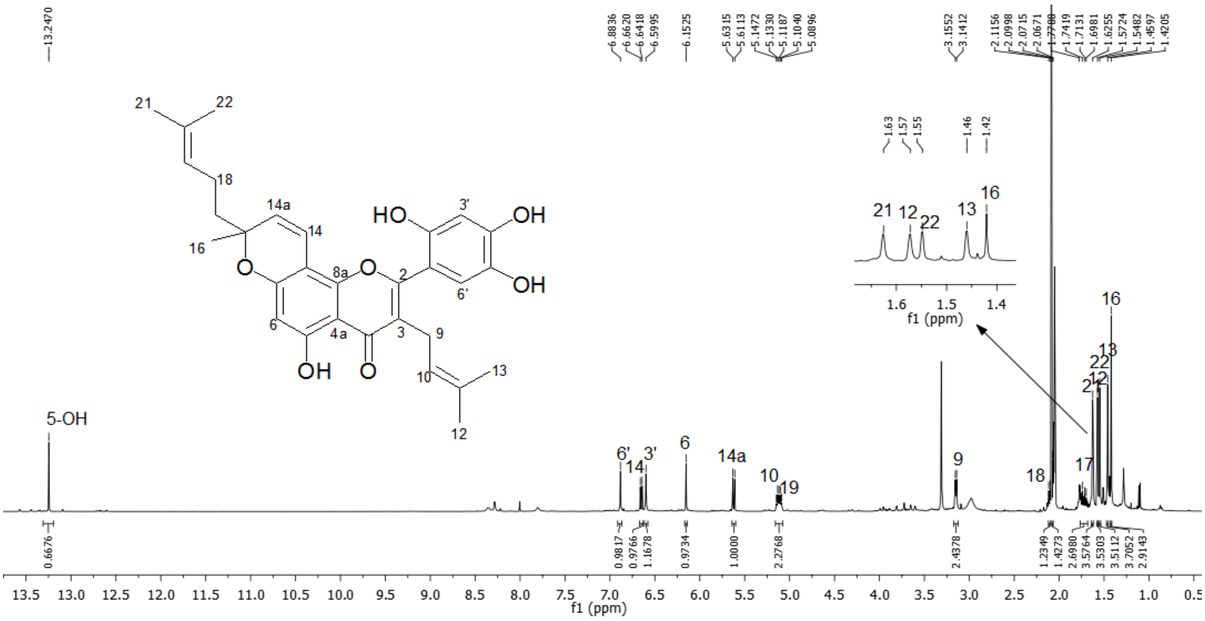

**Figure S8.** $^{1}$H-NMR spectrum of compound **5** (500mHz, acetone-$d_6$)

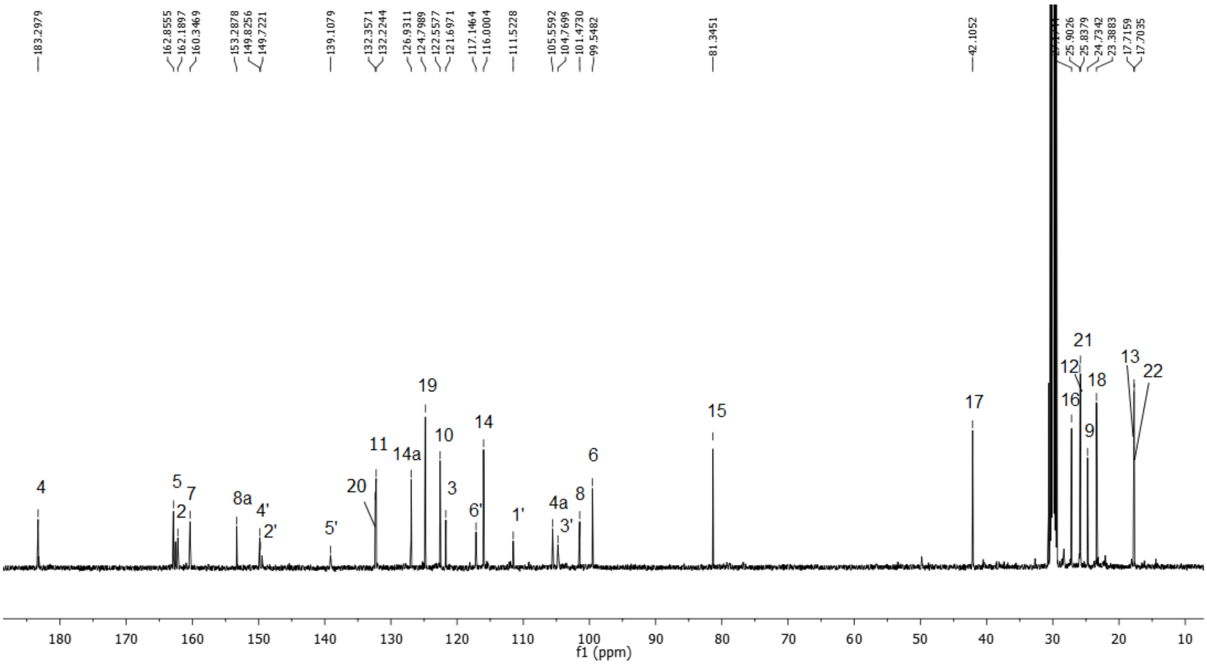

**Figure S9.** $^{13}$C-NMR spectrum of compound **5** (125mHz, acetone-$d_6$)

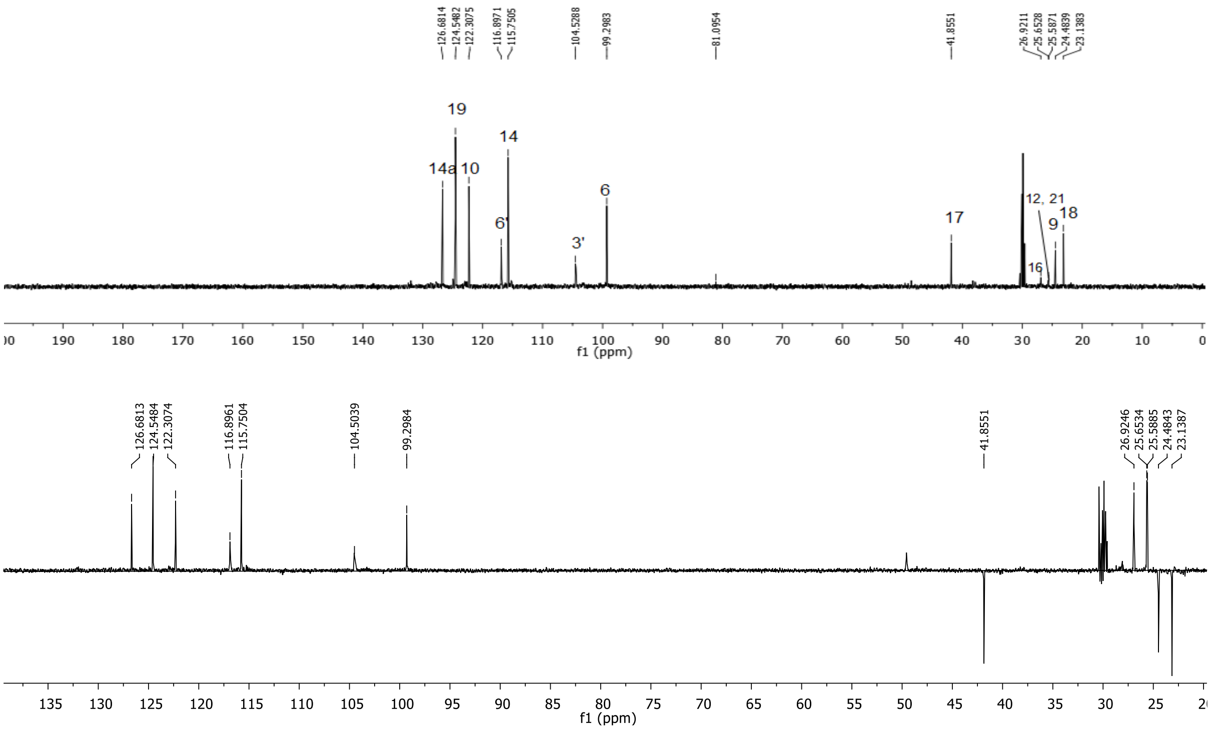


**Figure S10.** DEPT135 spectrum of compound **5**.

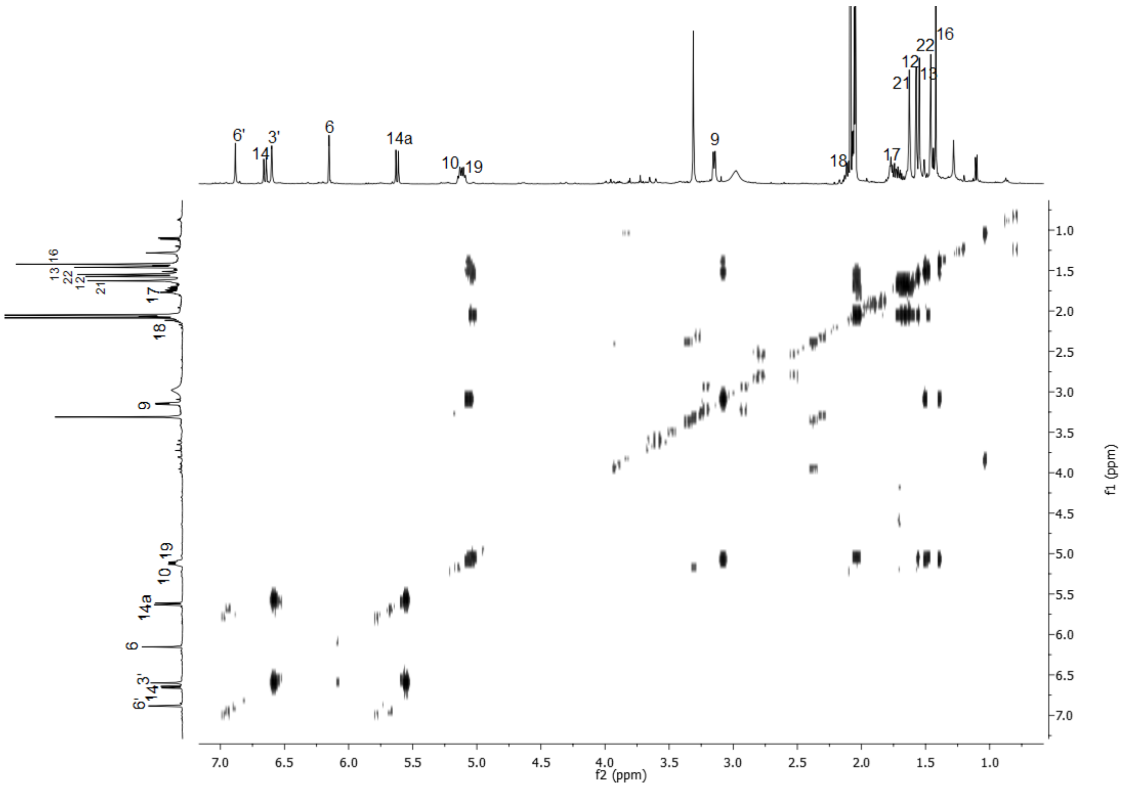


**Figure S11**. COSY spectrum of compound **5**.

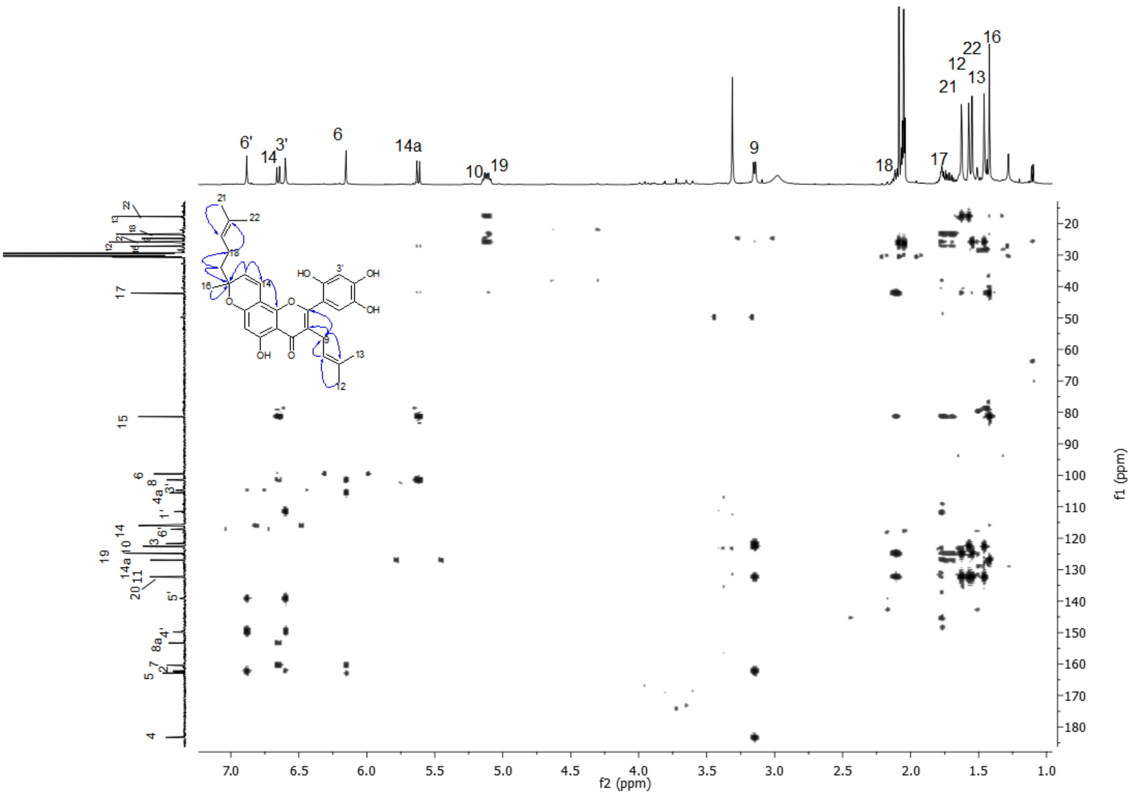


**Figure S12**. HMBC spectrum of compound **5**.

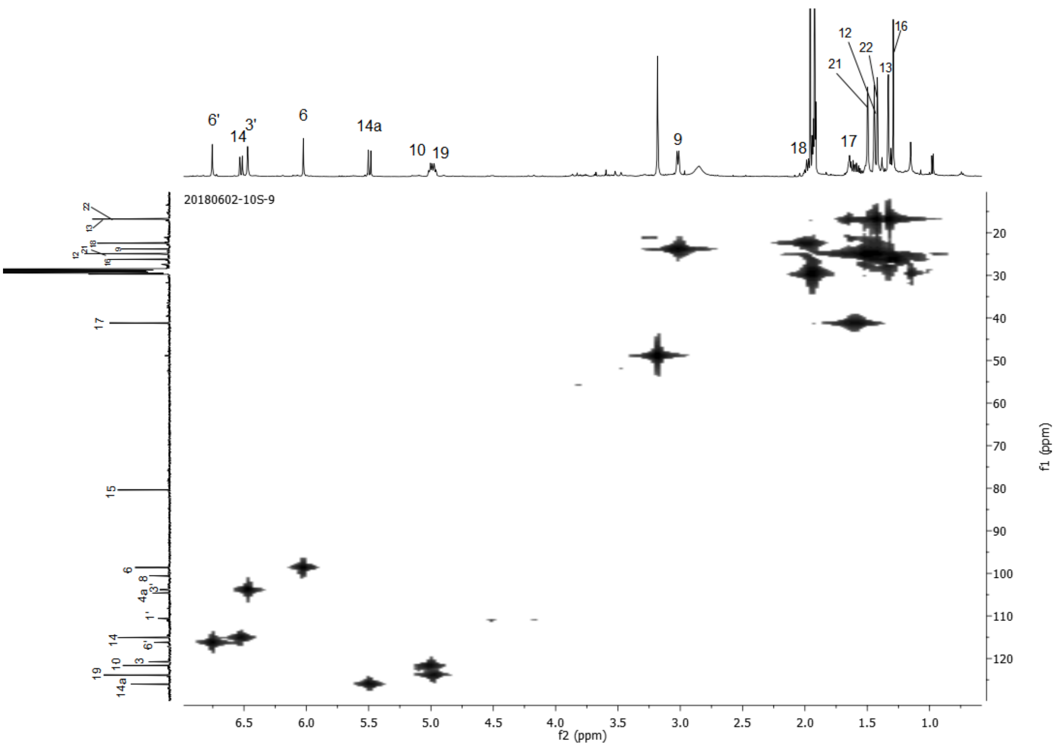


**Figure S13**.HMQC spectrum of compound **5**.

[ Mass Spectrum ]
Data : A10-S9 Date : 08-Jan-2019 15:52
Sample: -
Note : -
Inlet : Direct Ion Mode : FAB+
Spectrum Type : Normal Ion [MF-Linear]
RT : 0.30 min Scan# : 3
BP : m/z 154.1076 Int. : 54.73
Output m/z range : 100.0000 to 1002.0772 Cut Level : 0.00 %

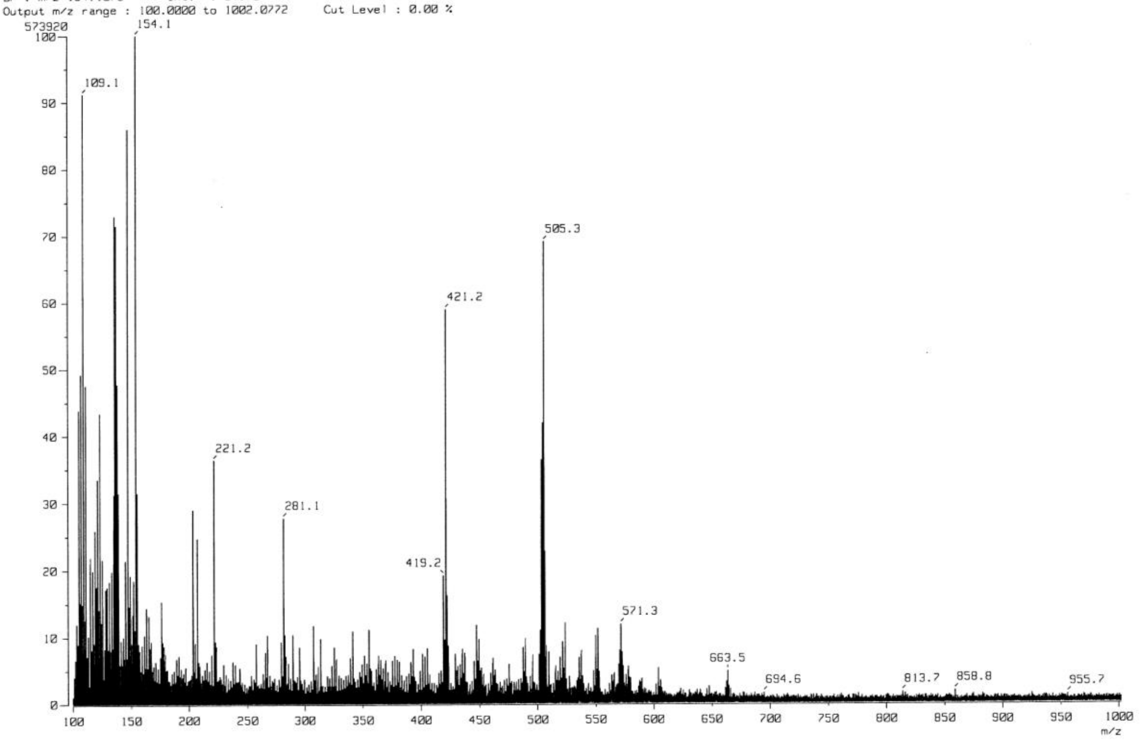


[ Elemental Composition ]
Data : A10-S9_HR Date : 08-Jan-2019 17:31
Sample: -
Note : -
Inlet : Direct Ion Mode : FAB+
RT : 4.84 min Scan#: 59
Elements : C 110/1, H 130/1, O 8/1
Mass Tolerance : 100ppm, 3mmu if m/z > 30
Unsaturation (U.S.) : 0.0 - 30.0

| Observed m/z | Int% | Err[ppm / mmu] | U.S. | Composition |
|---|---|---|---|---|
| 505.2216 | 34.6 | -1.9 / -1.0 | 14.5 | C 30 H 33 O 7 |

**Figure S14**. FABMS and HRFABMS data of compound **5**.

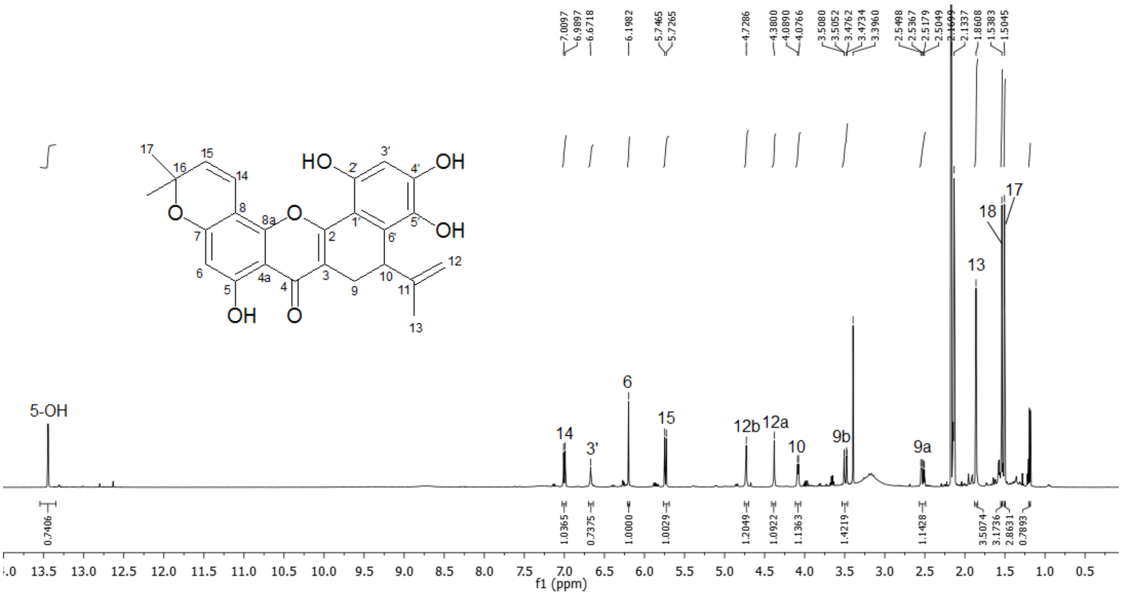


**Figure S15.** $^{1}$H-NMR spectrum of compound **2** (500mHz, acetone-$d_6$)

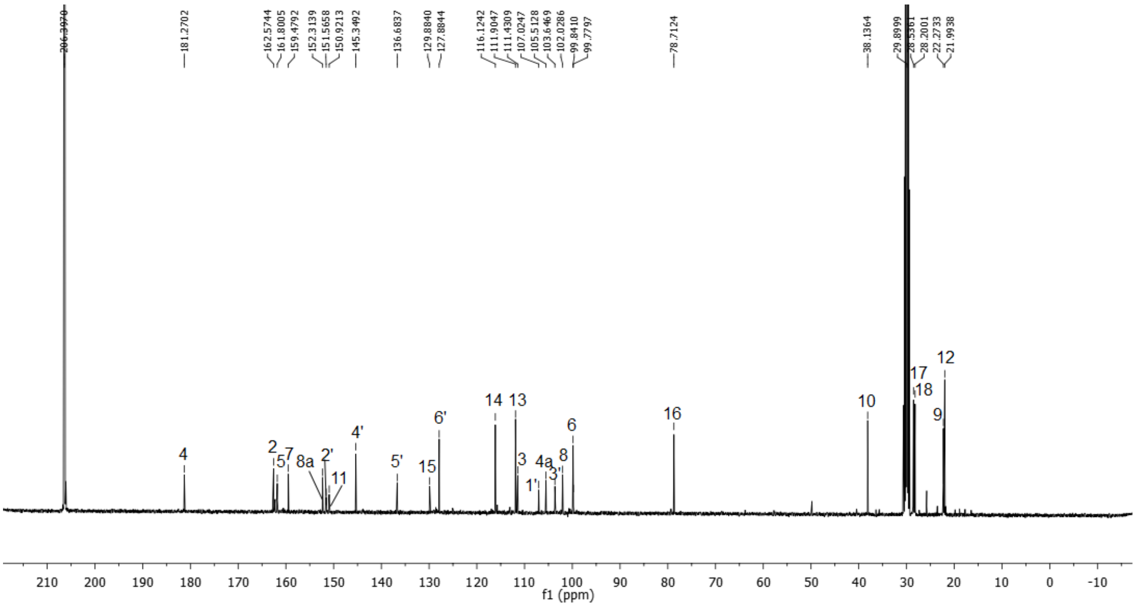


**Figure S16.** $^{13}$C-NMR spectrum of compound **2** (125mHz, acetone-$d_6$)

Data : A-F2_HR Date : 19-Jun-2018 14:03
Instrument : MStation
Sample : –
Note : –
Inlet : Direct Ion Mode : EI+
RT : 1.80 min Scan# : 28
Elements : C 100/1, H 100/1, O 10/1
Mass Tolerance : 1000ppm, 3mmu if m/z > 3
Unsaturation (U.S.) : -0.5 – 20.0

| | Observed m/z | Int% | Err[ppm / mmu] | U.S. | Composition |
|---|---|---|---|---|---|
| 1 | 434.1363 | 21.31 | -0.6 / -0.3 | 15.0 | C25 H22 O7 |

**Figure S17**. HR-ESI-MS data of compound **2**.

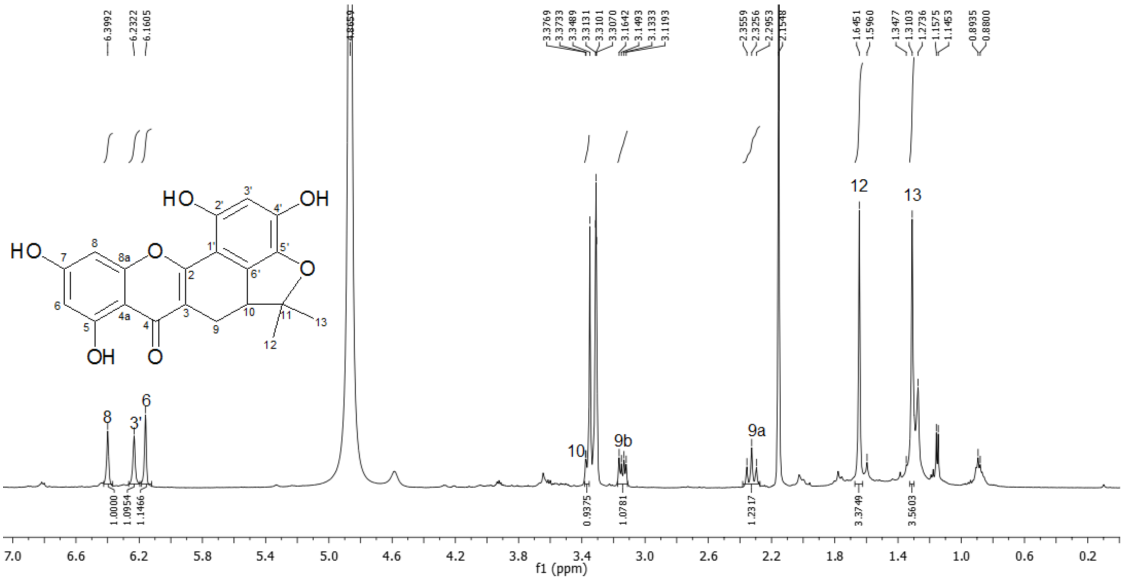


**Figure S18.** $^{1}$H-NMR spectrum of compound **3** (500mHz, acetone-$d_6$)

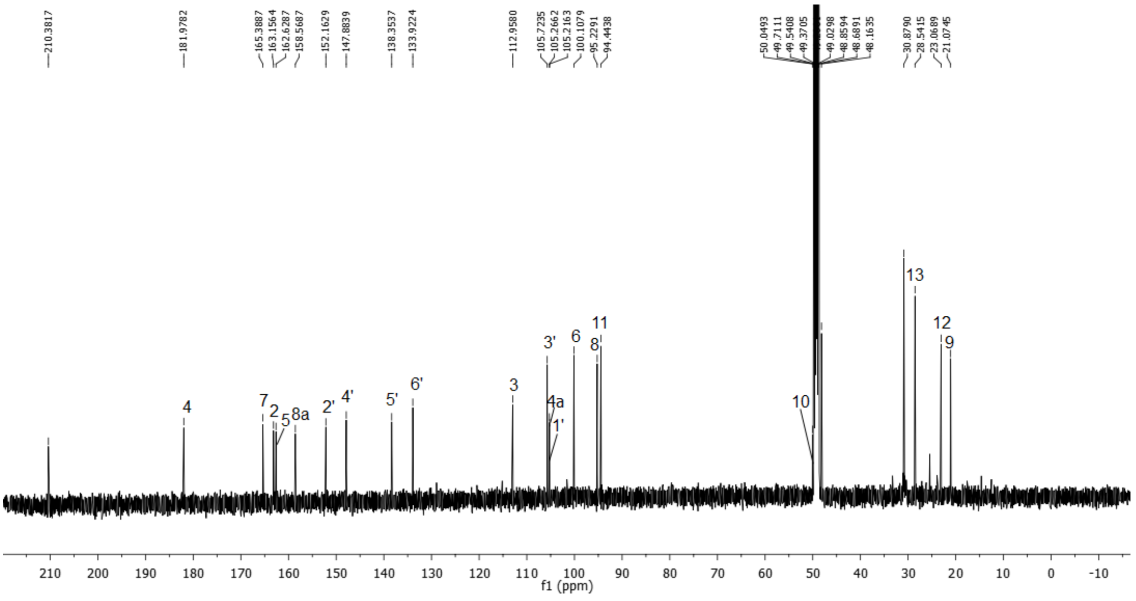


**Figure S19.** $^{13}$C-NMR spectrum of compound **3** (125mHz, acetone-$d_6$)

Data : F3-4_HR Date : 19-Jun-2018 15:31
Instrument : MStation
Sample : -
Note : -
Inlet : Direct Ion Mode : EI+
RT : 1.94 min Scan# : 30
Elements : C 100/1, H 100/1, O 10/1
Mass Tolerance : 1000ppm, 3mmu if m/z > 3
Unsaturation (U.S.) : -0.5 - 20.0

| | Observed m/z | Int% | Err[ppm / mmu] | U.S. | Composition |
|---|---|---|---|---|---|
| 1 | 368.0899 | 21.53 | +0.8 / +0.3 | 13.0 | C20 H16 O7 |

**Figure S20**. HR-ESI-MS data of compound **3**.

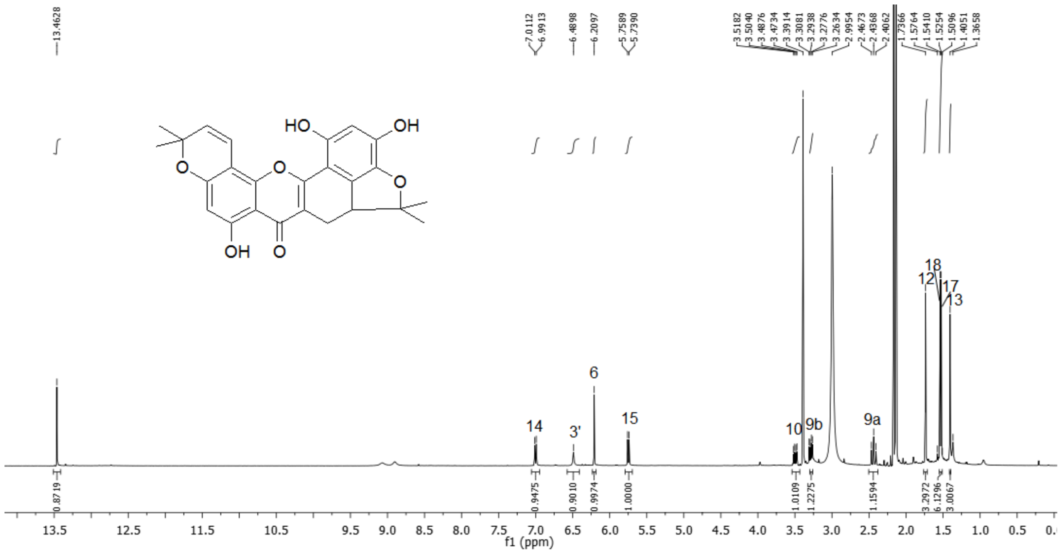


**Figure S21**. $^{1}$H-NMR spectrum of compound **4** (500mHz, acetone-$d_6$)

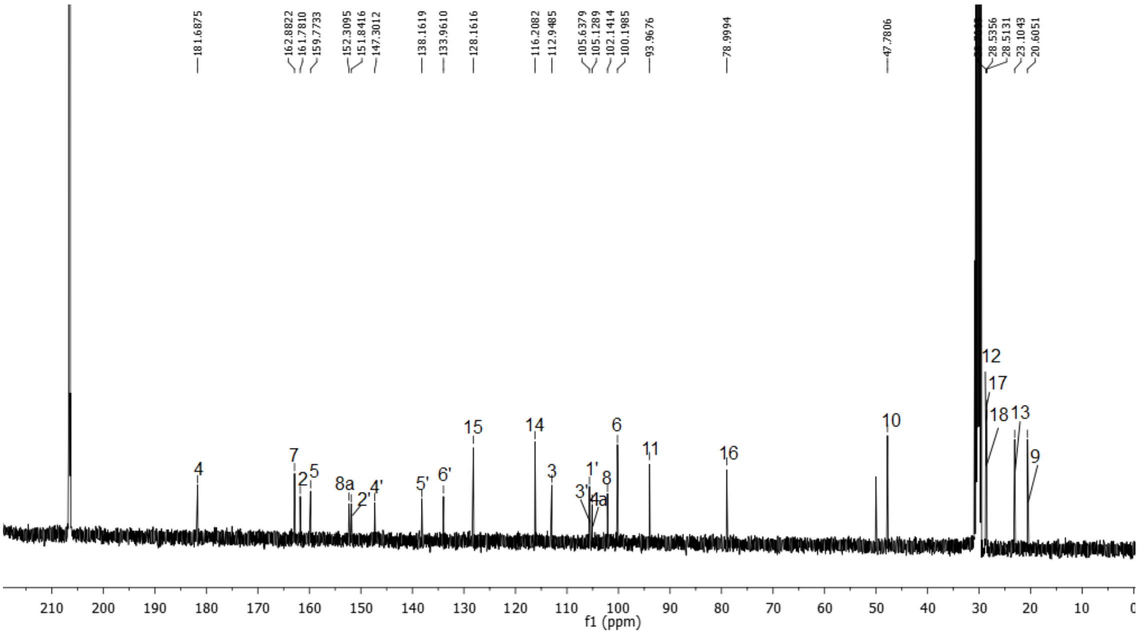


**Figure S22**. $^{13}$C-NMR spectrum of compound **4** (125mHz, acetone-$d_6$)

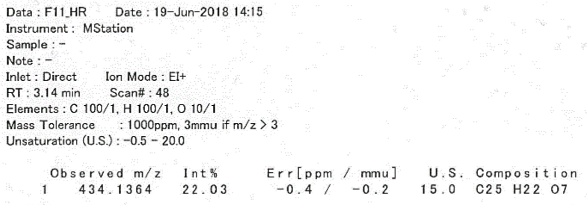

Data : F11_HR    Date : 19-Jun-2018 14:15
Instrument : MStation
Sample : -
Note : -
Inlet : Direct    Ion Mode : EI+
RT : 3.14 min    Scan# : 48
Elements : C 100/1, H 100/1, O 10/1
Mass Tolerance    : 1000ppm, 3mmu if m/z > 3
Unsaturation (U.S.) : -0.5 - 20.0

| | Observed m/z | Int% | Err[ppm / mmu] | U.S. | Composition |
|---|---|---|---|---|---|
| 1 | 434.1364 | 22.03 | -0.4 / -0.2 | 15.0 | C25 H22 O7 |

**Figure S23**. HR-ESI-MS data of compound **4**.

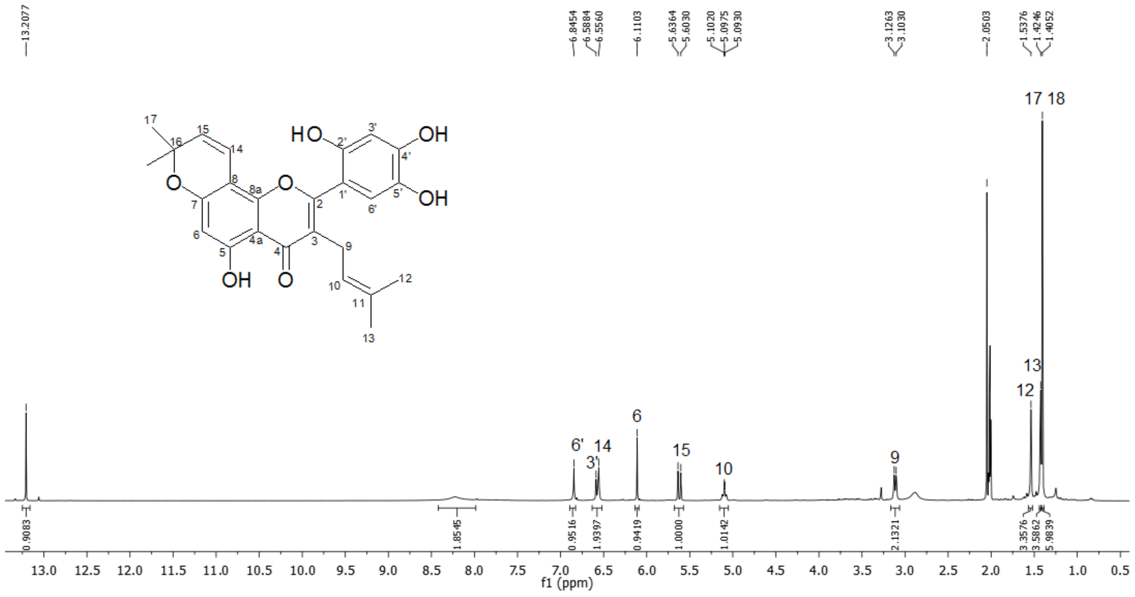


**Figure S24**. $^{1}$H-NMR spectrum of compound **6** (500mHz, acetone-$d_6$)

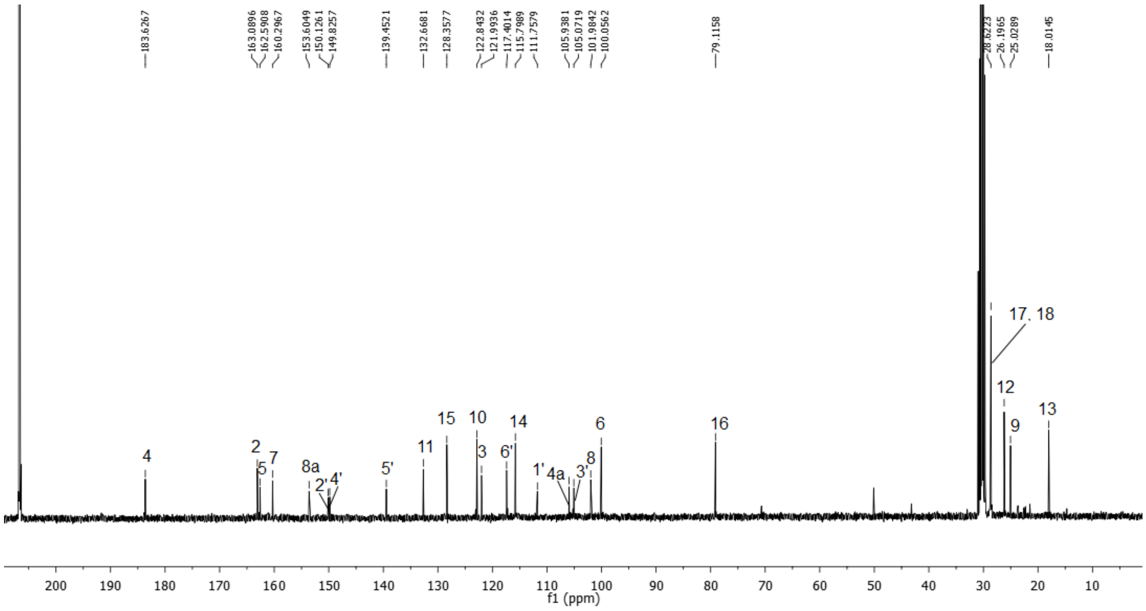


**Figure S25**. $^{13}$C-NMR spectrum of compound **6** (125mHz, acetone-$d_6$)

```
[ Elemental Composition ]                                        P
Data   : A-F8_HR                     Date : 08-Jan-2019 17:22
Sample: -
Note : -
Inlet : Direct                       Ion Mode : FAB+
RT : 0.59 min                        Scan#: 8
Elements : C 110/1, H 130/1, O 8/1
Mass Tolerance      : 100ppm, 5mmu if m/z > 50
Unsaturation (U.S.) : 0.0 - 30.0

Observed m/z  Int%    Err[ppm / mmu]    U.S.  Composition
  437.1635    20.4      +7.8 /  +3.4    13.5  C 25 H 25 O 7
```

**Figure S26**. HR-ESI-MS data of compound **6**.

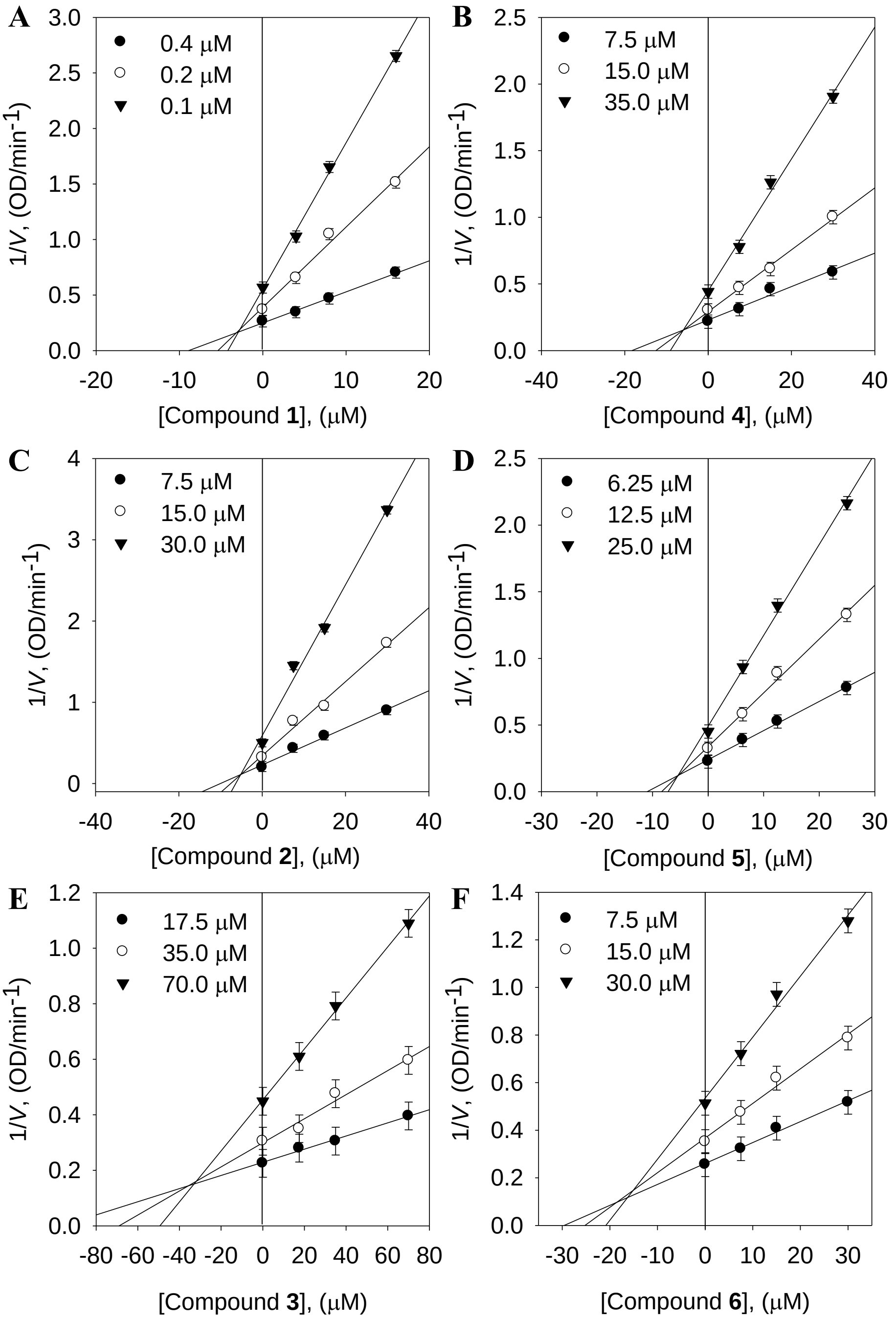


**Figure S27.** Dixon plots for α-glucosidase of compounds **1-6**.

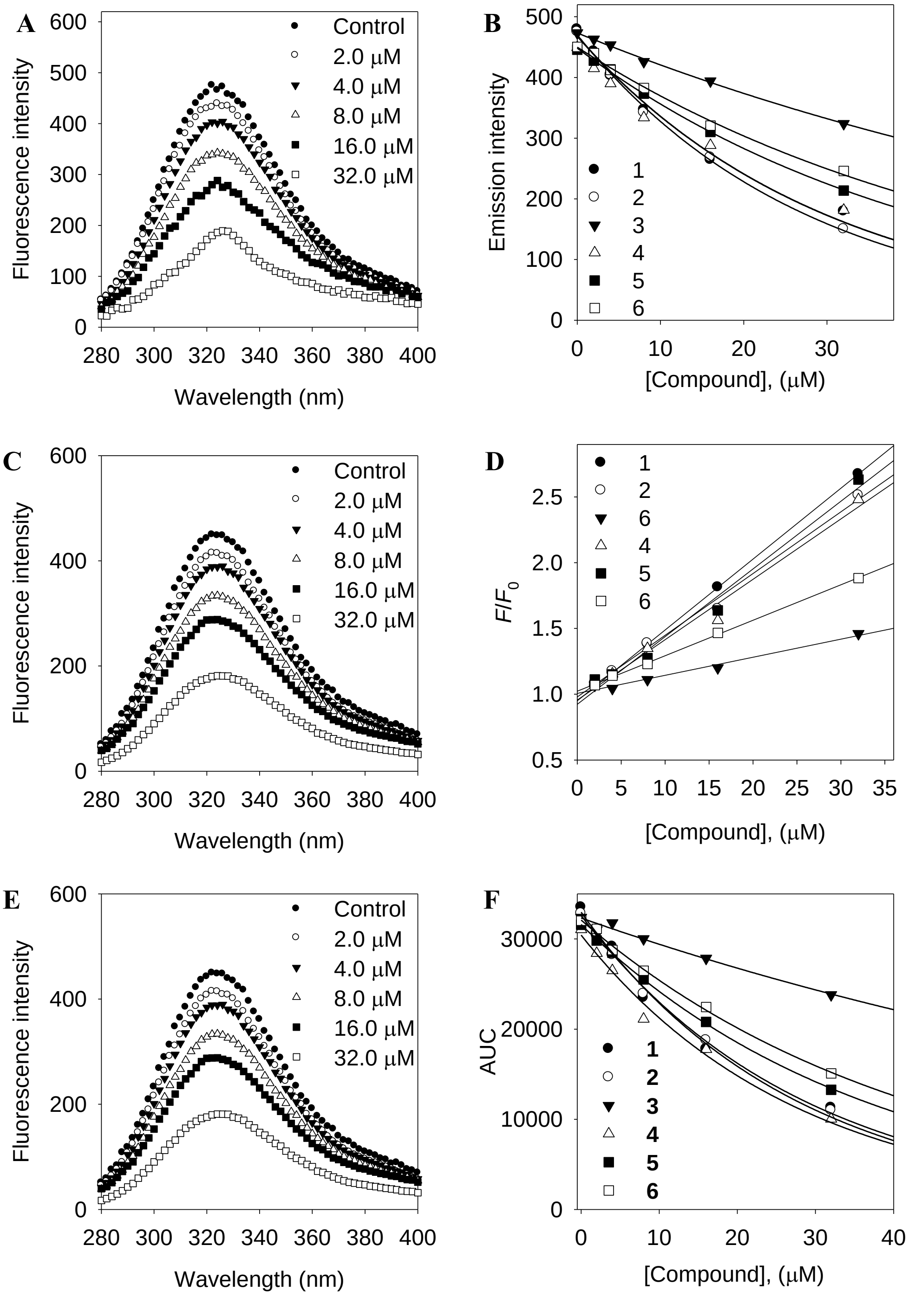


**Figure S28.** (A) The fluorescence effect of compound **2**, (C) compound **4** and (E) compound **5** on fluorescence emission spectra of α-glucosidase, (B) Decrease in intensity of the emission plots of compounds, (D) Stern-Volmer plots of compounds at 37 °C, (F) Area under curve (AUC) of compounds.

```
  1 MTISDHPETE PKWWKEATIY QIYPASFKDS NNDGWGDLKG ITSKLQYIKD
 51 LGVDAIWVCP FYDSPQQDMG YDISNYEKVW PTYGTNEDCF ELIDKTHKLG
101 MKFITDLVIN HCSTEHEWFK ESRSSKTNPK RDWFFWRPPK GYDAEGKPIP
151 PNNWKSFFGG SAWTFDETTN EFYLRLFASR QVDLNWENED CRRAIFESAV
201 GFWLDHGVDG FRIDTAGLYS KRPGLPDSPI FDKTSKLQHP NWGSHNGPRI
251 HEYHQELHRF MKNRVKDGRE IMTVGEVAHG SDNALYTSAA RYEVSEVFSF
301 THVEVGTSPF FRYNIVPFTL KQWKEAIASN FLFINGTDSW ATTYIENHDQ
351 ARSITRFADD SPKYRKISGK LLTLLECSLT GTLYVYQGQE IGQINFKEWP
401 IEKYEDVDVK NNYEIIKKSF GKNSKEMKDF FKGIALLSRD HSRTPMPWTK
451 DKPNAGFTGP DVKPWFLLNE SFEQGINVEQ ESRDDDSVLN FWKRALQARK
501 KYKELMIYGY DFQFIDLDSD QIFSFTKEYE DKTLFAALNF SGEEIEFSLP
551 REGASLSFIL GNYDDTDVSS RVLKPWEGRI YLVK
```

**Figure S29.** Amino acid sequence of *Saccharomyces cerevisiae* α-glucosidase. The Trp(W), Phe(F), and Tyr(Y) residues are shown as yellow, cyan, and pink respectively.

Trp(W): 20, Phe(F): 41, Tyr(Y): 26

**Table S1**. $^1H$ and $^{13}C$ spectroscopic data of ($\delta$ in ppm, $J$ in Hz) **1** and **5** (acetone-$d_6$)

| No. | **1** | | **5** | |
|---|---|---|---|---|
| | $\delta_H$ $J$ (Hz) | $\delta_C$ m | $\delta_H$ $J$ (Hz) | $\delta_C$ m |
| 2 | | 161.9 | | 162.2 |
| 3 | | 111.7 | | 121.7 |
| 4 | | 181.1 | | 183.3 |
| 4a | | 105.6 | | 105.6 |
| 5 | | 162.9 | | 162.8 |
| 6 | 6.29 d (2.25) | 98.7 | 6.15 s | 99.6 |
| 7 | | 166.1 | | 160.4 |
| 8 | 6.65 d (2.25) | 93.2 | | 101.5 |
| 8a | | 157.7 | | 153.2 |
| 9a | 2.43 dd (15.98) | 22.4 | 3.14 d (7) | 24.7 |
| 9b | 3.39 d (15.95) | | | |
| 10 | 3.99 d (6.3) | 38.0 | 5.09 m | 122.6 |
| 11 | | 145.4 | | 132.2 |
| 12 | 1.78 s | 22.0 | 1.57 s | 25.9 |
| 13a | 4.29 s | 111.9 | 1.46 s | 17.7 |
| 13b | 4.65 s | | | |
| 14 | | | 5.61 d (10.1) | 116.0 |
| 14a | | | 5.61 d (10.1) | 126.9 |
| 15 | | | | 81.4 |
| 16 | | | 1.42 s | 27.2 |
| 17 | | | 1.69 m | 42.1 |
| 18 | | | 2.07 m | 23.4 |
| 19 | | | 5.09 m | 124.8 |
| 20 | | | | 132.4 |
| 21 | | | 1.63 s | 25.8 |
| 22 | | | 1.54 s | 17.7 |
| 1' | | 106.6 | | 111.5 |
| 2' | | 151.4 | | 149.7 |
| 3' | 6.50 s | 103.8 | 6.59 s | 104.8 |
| 4' | | 151.2 | | 149.8 |
| 5' | | 136.9 | | 139.1 |
| 6' | | 129.4 | 6.88 s | 117.2 |
| OMe | 3.89 s | 56.4 | | |

**Table S2.** Determination of $K_{ik}/K_{iv}$ ratios by α-glucosidase enzyme inhibitory behaviours.

| Compounds | [I] (μM) | $V_{max}$ | $K_m$ | $K_{ik}/K_{iv}$ |
|---|---|---|---|---|
| **2** | 0 | 9.337 | 186.0878 | - |
| | 7.5 | 9.7056 | 740.5780 | 76.6587 |
| | 15 | 9.6712 | 868.3559 | 102.4604 |
| | 30 | 9.8012 | 2117.5032 | 209.3396 |
| **3** | 0 | 6.5274 | 193.6945 | - |
| | 17.5 | 6.6800 | 301.7435 | 23.8593 |
| | 35 | 6.7889 | 439.0428 | 31.6240 |
| | 70 | 6.7204 | 627.7285 | 75.7804 |
| **4** | 0 | 6.8027 | 202.3061 | - |
| | 7.5 | 6.3816 | 397.2304 | 16.6987 |
| | 15 | 7.3692 | 812.0855 | 11.4837 |
| | 30 | 7.4627 | 1318.5821 | 20.2662 |
| **6** | 0 | 6.8013 | 194.8085 | - |
| | 7.5 | 5.0352 | 265.5740 | 3.7049 |
| | 15 | 4.2992 | 319.4798 | 2.5098 |
| | 30 | 3.6219 | 365.4835 | 1.8101 |
